# Effects of thermal cycling and room-temperature ageing on bismuth precipitates in Sn-Ag-Cu-Bi solder joints

**DOI:** 10.1007/s10853-026-12718-8

**Published:** 2026-04-28

**Authors:** C. L. Hsieh, R. J. Coyle, J. W. Xian, C. M. Gourlay

**Affiliations:** 1 Department of Materials, Imperial College London, London, SW7 2AZ UK; 2 Nokia Bell Labs, Murray Hill, NJ USA; 3 School of Materials Science and Engineering, Dalian University of Technology, Dalian, China

## Abstract

The long-term stability of Bi-strengthened Sn–Ag–Cu solders is an important but insufficiently understood factor for the reliability of stored electronic assemblies. In this study, (Bi) precipitates in Sn-2.25Ag-0.5Cu-6Bi (wt.%) solder joints were examined in two ball grid array (BGA) packages after long-term (> 6 years) room-temperature ageing following reflow soldering and after harsh thermal cycling between − 55 and 125 °C. Significant differences are observed between bulk and surface (Bi) precipitates in their distribution, morphology, crystallographic orientation relationships (ORs) with *β*-Sn, and the formation of interfaces and ORs with Cu₆Sn₅. Three distinct ORs between (Bi) and *β*-Sn are identified, each with eight crystallographic variants in good agreement with calculated variants. The precipitates are plate-shaped, with the largest habit planes corresponding to the most coherent interfaces (${\{100\}}_{\beta \mathrm{Sn}}\parallel {\{01\bar{1}2\} }_{\left(\mathrm{Bi}\right)}$ for OR1; ${\{011\}}_{\beta \mathrm{Sn}}\parallel {\{01\bar{1}2\} }_{\left(\mathrm{Bi}\right)}$ for OR2), resulting in multiple precipitate alignments within the matrix. Notably, OR2 is preferentially observed for bulk precipitates. Two additional ORs are identified between (Bi) and Cu₆Sn₅. After prolonged room-temperature storage, however, the bulk (Bi) precipitates become coarsened and widely spaced (> 1 μm in size and > 9 μm spacing), and (Bi) is observed to accumulate adjacent to Cu₆Sn₅ and Ag₃Sn particles. These observations indicate over-ageing and significant microstructural evolution even at room temperature, suggesting a potential long-term reliability concern for Bi-strengthened solder joints in stored electronics.

**Graphical abstract:**

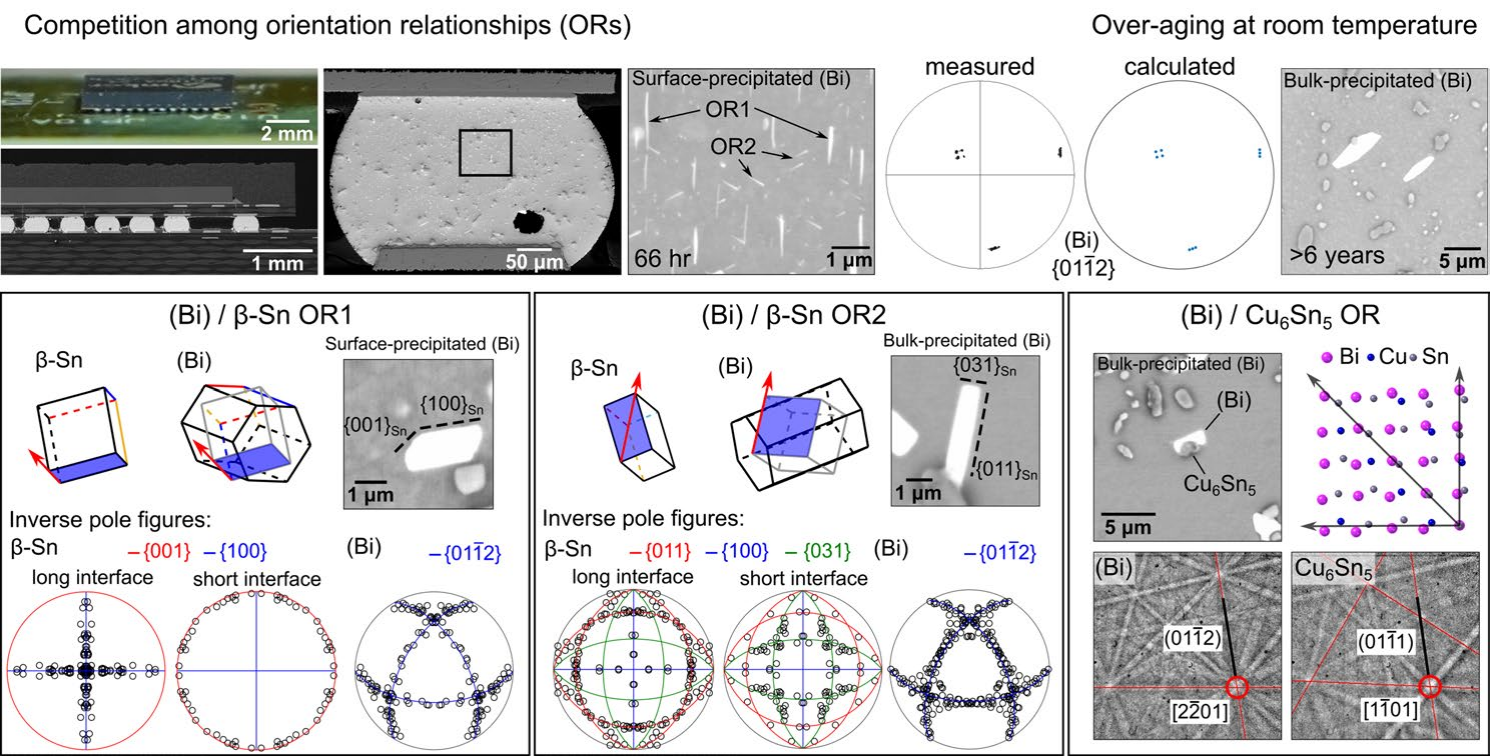

**Supplementary Information:**

The online version contains supplementary material available at 10.1007/s10853-026-12718-8.

## Introduction

Sn–Ag–Cu–Bi solder alloys containing ~ 3–6 wt.% Bi are attractive as high reliability solders [1], for components in automotive, aerospace and defence applications with long operational life requirements and higher consequences of failure. For these applications under harsh environments, thermal fatigue is a major issue, which results from the thermal expansion mismatch between electronic components and the printed circuit board (PCB) and between the grains and phases in the solder microstructure [2, 3]. Under mild thermal cycling, such as 0/100 °C, Sn–Ag–Cu (SAC) solders such as Sn-3Ag-0.5Cu usually exhibit good thermal fatigue life due to adequate particle strengthening by eutectic Ag_3_Sn particles [4, 5]. However, under harsher thermal cycling such as −40/125 °C or −55/125 °C, the Ag_3_Sn strengthening effect diminishes as the Ag_3_Sn particles coarsen under high temperature and plastic strain [6, 7] as quantified in reference [6]. To further improve reliability, a range of Sn–Ag–Cu-based solders with additions of 3–6 wt.% of Sb, Bi, and/or In has been developed, [1, 8–10]. The design strategy is to provide (i) solid solution strengthening by the dissolved atoms in *β*-Sn [1, 11, 12] and (ii) particle strengthening. In the case of Sn–Ag–Cu–Bi solders of sufficient Bi concentration, the latter is by the (Bi) phase which precipitates in the solid state from supersaturated *β*-Sn [1, 8, 11–13].

Bi-containing solders are of particular interest since Bi reduces the liquidus temperature (e.g. adding 6 wt.% Bi to Sn-2.25Ag-0.5Cu-xBi lowers the *β*-Sn liquidus by ~ 8 K compared with Sn-2.25Ag-0.5Cu—see SI-Fig. 1 in the Supplementary Information) [14, 15], improves wettability [14, 16, 17], suppresses interfacial intermetallic compound (IMC) layer growth [16, 18, 19], and prevents tin whiskers [20, 21] in lead-free solders. Previous research has demonstrated that Bi enhances mechanical properties including shear strength [14, 22–26], tensile strength [11, 12, 14, 27–30], and creep resistance [15, 18, 26, 31, 32], and that these collectively improve thermal cycling reliability in many cases [8, 25, 33–36]. However, some studies (e.g. [37]) have reported relatively poor thermal fatigue performance in Sn–Ag–Cu–Bi solders containing (Bi) precipitates and, therefore, further research is required to understand microstructure evolution in these solders.

In past studies on as-solidified solders, the (Bi) phase was observed in Sn-3.8Ag-0.7Cu-4Bi [38], Sn-2.5Ag-0.7Cu-2.5Bi [39], Sn-1.5Ag-0.7Cu-3Bi [15], and Sn-0.6Ag-0.7Cu-5Bi [40] (wt.%). In contrast, the (Bi) phase was not reported in Sn-3.8Ag-0.7Cu-2Bi [38], Sn-2.5Ag-0.7Cu-1Bi [39], Sn-1.5Ag-0.7Cu-1Bi [15], and Sn-0.6Ag-0.7Cu-2.5Bi [40] (wt.%). From this, it may seem that ~ 2.5 wt.% Bi is required to have (Bi) precipitation from *β*-Sn in Sn–Ag–Cu–Bi alloys. However, the presence or absence of (Bi) precipitation also depends on the prior thermal history. For example, after solidification, the *β*-Sn will be highly supersaturated in Bi near the non-equilibrium (Bi) eutectic due to microsegregation during solidification, resulting in some solid-state (Bi) precipitation in these regions even for an alloy with Bi concentration below 3 wt.% [39, 41, 42]. In contrast, ageing or thermal cycling will lead to some degree of solutionising and homogenisation and bring the alloy closer towards equilibrium. For example, in a recent study that applied 4876 thermal cycles from −55/125 °C to SAC + Bi solder joints [37], no (Bi) precipitates were found in Sn-3Ag-0.8Cu-3.0Bi and Sn-4Ag-0.6Cu-3.5Bi solder joints, and copious (Bi) precipitation occurred in Sn-2.25Ag-0.5Cu-6.0Bi (wt.%) after thermal cycling. From this, Sn–Ag–Cu–Bi solders can be categorised into those with approximately < 4 wt.% Bi that contain little or no (Bi) phase after an initial period of ageing or thermal cycling where the Bi mostly provides solid solution strengthening, and those containing > 4 wt.% Bi that are additionally (Bi) precipitation strengthened. Most studies in the literature have focussed on the first category with < 4 wt.% Bi [15, 38, 43–47]. While there have been studies on Sn–Ag–Cu–Bi solders with > 4 wt.% Bi [40, 48, 49], much of the detail of (Bi) precipitates and their evolution during long-term storage remains to be explored.

In cases where solid-state (Bi) precipitation occurs, aligned (Bi) precipitates have been observed in several previous studies [33, 39, 50–53]. Belyakov et al. [51] investigated the crystallography of solid-state-precipitated (Bi) phase in Sn–Ag–Cu–Bi joints using Sn-1Ag-0.5Cu-4Bi and Sn-3Ag-0.5Cu-4Bi. Most of the (Bi) precipitates were well-aligned in one direction, and EBSD revealed the orientation relationship OR1 between these (Bi) particles and the *β*-Sn matrix:

OR1 $$\left\{ {100} \right\}_{{\beta {\mathrm{Sn}}}} \parallel \left\{ {01\bar{1}2} \right\}_{{\left( {{\mathrm{Bi}}} \right)}} {\text{and }}\left\langle {100} \right\rangle _{{\beta {\mathrm{Sn}}}} \parallel \left\langle {2\bar{2}01} \right\rangle _{{\left( {{\mathrm{Bi}}} \right)}}$$

Through combined electron backscattered diffraction (EBSD) and focused ion beam (FIB) tomography on (Bi) particles with OR1, it was deduced that the particles were elongated along ${\langle 001\rangle }_{\beta \mathrm{Sn}}$ and the particles had two common habit planes, ${(100)}_{\beta \mathrm{Sn}}{\parallel (01\bar{1 }2)}_{\left(\mathrm{Bi}\right)}$ and ${(010)}_{\beta \mathrm{Sn}}{\parallel (\bar{1 }012)}_{\left(\mathrm{Bi}\right)}$. There were also a minority of (Bi) precipitates aligned in other directions, and OR2 was observed between most of these (Bi) precipitates and the *β*-Sn matrix. During room-temperature ageing, (Bi) particles with OR1 were more stable and grew at the expense of (Bi) particles with OR2.

OR2 $$\left\{ {100} \right\}_{{\beta {\mathrm{Sn}}}} \parallel \left\{ {01\bar{1}2} \right\}_{{\left( {{\mathrm{Bi}}} \right)}} {\text{and }}\left\langle {011} \right\rangle _{{\beta {\mathrm{Sn}}}} \parallel \left\langle {2\bar{2}01} \right\rangle _{{\left( {{\mathrm{Bi}}} \right)}}$$

These findings [51] were obtained from precipitation on a free surface after polishing, and the surface kinetics were noted to be more rapid than in the bulk in [51]. Additionally, Wu et al. [41] reported that (Bi) preferentially precipitates on polished surfaces and Hsieh et al. [53] showed that the area fraction of (Bi) precipitates on polished surfaces increases to several times the equilibrium volume fraction of (Bi) during room-temperature ageing. Thus, there is a need for a new study to compare (Bi) precipitation mechanisms in the bulk volume versus on a polished surface. Also, since room temperature is a homologous temperature of ~ 0.6 T_*m*_ for Sn–Ag–Cu–Bi solders and electronics can be stored for years before or between uses, further information is required on the microstructure stability of bulk (Bi) precipitates during room-temperature storage over a period of years.

A further factor that has not been addressed in past work on Sn–Ag–Cu–Bi solders is the crystallographic orientation variants in the ORs. In other alloy systems including steels [54], superalloys [55], aluminium alloys [56], and magnesium alloys [57], orientation relationships with multiple orientation variants often lead to precipitates with multiple different alignments in the matrix which can be important for strengthening. To study crystallographic orientation variants in Sn–Ag–Cu–Bi solder alloys and how they are affected by thermal history, the study by Belyakov et al. [51] needs to be expanded. For example, the role of the rhombohedral structure of (Bi) with ${\left\{01\bar{1}2\right\} }_{\left(\mathrm{Bi}\right)}$ planes that are ~ 87.5° apart needs to be considered when interpreting habit planes and precipitate morphologies, the ORs between (Bi) and intermetallic phases (Cu_6_Sn_5_ and Ag_3_Sn) need to be elucidated, and precipitation during room-temperature storage needs to be compared with (Bi) precipitation and coarsening in solder joints that experience the temperature cycling conditions of accelerated thermal cycling (ATC).

To address these questions, this paper explores the crystallographic aspects of (Bi) precipitation in Sn-2.25Ag-0.5Cu-6Bi solder joints in two ball grid array (BGA) electronic test vehicles subjected to two thermal histories: (i) reflow soldering followed by ~ 7 years room-temperature storage, and (ii) reflow soldering followed by harsh accelerated thermal cycling (ATC) of 4876 temperature cycles from −55 to 125 °C, followed by ~ 6 years room-temperature storage. Our approach couples scanning electron microscopy (SEM) and electron backscatter diffraction (EBSD) analyses to explore the morphologies, orientation variants, and habit planes of (Bi) precipitates with each OR, and explores ORs between (Bi) precipitates and IMC phases for both precipitation on the free surface and precipitation in the bulk solder.

## Methods

Sn-2.25Ag-0.5Cu-6Bi (wt.%) solder balls, 460 μm or 300 μm in diameter, and paste of the same composition were used to solder 192CABGA or 84CTBGA ball grid array (BGA) packages, respectively, to a printed circuit board (PCB) as described in detail in reference [1]. Some of the as-soldered test vehicles were stored at room temperature (23 ± 2 °C) for 7.2 years before characterisation. This thermal history is designated as “soldered-then-stored” in this paper. The remaining test vehicles were thermally cycled from −55 to 125 °C at a cooling and heating rate of 10 K/min for each cycle, with a 10 min dwell time at the hot and cold ends of the cycle, i.e. each cycle lasted 56 min involving heating from −55 to 125 °C in 18 min, holding at 125 °C for 10 min, cooling to −55 °C in 18 min and holding at −55 °C for 10 min. The thermally cycled packages studied here had each experienced 4876 cycles. These test vehicles were then stored at room temperature (23 ± 2 °C) for 6.4 years before characterisation. This thermal history is designated as “thermally-cycled-then-stored” in this paper.

For characterisation, one package for each condition (thermal history + package type) was cut from the PCB, mounted in Struers VersoCit II cold mounting resin, wet ground to 4000 grit SiC paper to near the centre of a row of joints, polished with colloidal silica and then coated with ~ 3 nm of carbon. For each package, 12–20 solder joints were examined. Analytical scanning electron microscopy (SEM) was conducted on a Zeiss SIGMA field-emission gun SEM (Carl Zeiss, Oberkochen, Germany) with a Bruker e-FlashHR electron backscatter diffraction (EBSD) detector (Bruker AXS Inc., Fitchburg, WI, USA). Both backscattered electron (BSE) imaging and EBSD acquisition were performed at an accelerating voltage of 20 keV with a 120 µm aperture size. The working distance was ~ 9 mm for BSE imaging and ~ 15 mm for EBSD acquisition. For EBSD, the sample stage was tilted to 70°, and the acquisition step size ranged from 0.2 to 0.3 μm. EBSD patterns were indexed in Bruker Esprit 2.1 assuming the phases in Table 1. The confidence of the assigned orientations was assessed using the band mismatch (BMM), which describes the mean angular deviation between the detected bands and the corresponding theoretical bands. In this work, a BMM threshold of 1.5° and > 5 detected bands were used when processing orientation maps.

**Table 1:** Crystal structure information used for EBSD indexing and analyses in this study. FCR = face-centred rhombohedral non-standard unit cell

Phase	Lattice system	Space group	Cell setting	a [Å]	b [Å]	c [Å]	*α* [°]	*β* [°]	*γ* [°]	Ref
*β*-Sn	Tetragonal	I4_1_/amd		5.831	5.831	3.181	90	90	90	[62]
Ag_3_Sn	Orthorhombic	Pmmn		4.782	5.998	5.164	90	90	90	[63]
*η*-Cu_6_Sn_5_	Hexagonal	P6_3_/mmc		4.192	4.192	5.037	90	90	120	[64]
*η*${\prime}$-Cu_6_Sn_5_	Monoclinic	C2/c		11.022	7.282	9.827	90	98.84	90	[65]
(Bi)	Rhombohedral	R-3 m	Hexagonal	4.546	4.546	11.862	90	90	120	[66]
			FCR	6.572	6.572	6.572	87.536	87.536	87.536	

EBSD maps were analysed within Bruker Esprit 2.1, and EBSD Kikuchi patterns were further indexed with EDAX OIM to verify orientation relationships. The measured Euler angles were input into the MTEX toolbox in MATLAB to analyse the measured orientation variants of *β*-Sn/(Bi) ORs, to calculate theoretical variant orientations, and to calculate angles between the X, Y, Z sample coordinate system and the crystal coordinate system.

Examining Table 1, note that the rhombohedral (Bi) phase is listed in two unit-cell settings: a hexagonal cell and a face-centred rhombohedral (FCR) unit cell. The standard hexagonal unit cell was used for EBSD indexing. Both the hexagonal unit cell and the non-standard FCR unit cell are used in this paper to present and explain the orientation variants for OR1 and OR2. The FCR unit cell has a rhombohedral angle ~ 87.536°, which is pseudo-cubic with ~ 2.5° rhombohedral distortion [58, 59]. The FCR unit cell is enclosed by the six $\left\{ {01\bar{1}2} \right\}$ planes in hexagonal indices which are the closest packed planes in (Bi), and the $\langle 100\rangle$ axes of the FCR unit cell are the $\langle 2\bar{2 }01\rangle$ axes in the hexagonal cell setting. The transformation from hexagonal to FCR axes can be calculated using [60]:

$$\left( {\begin{array}{*{20}c} {a_{{{\mathrm{FCR}}}} } \\ {b_{{{\mathrm{FCR}}}} } \\ {c_{{{\mathrm{FCR}}}} } \\ \end{array} } \right) = \frac{1}{3}\left( {\begin{array}{*{20}c} { - 4} & { - 2} & 1 \\ 2 & { - 2} & 1 \\ 2 & 4 & 1 \\ \end{array} } \right)\left( {\begin{array}{*{20}c} {a_{{\mathrm{h}}} } \\ {b_{{\mathrm{h}}} } \\ {c_{{\mathrm{h}}} } \\ \end{array} } \right)$$

Under this transformation, the planes ${\left(\bar{1 }012\right)}_{\mathrm{h}}$, ${\left(1\bar{1 }02\right)}_{\mathrm{h}}$, ${\left(01\bar{1 }2\right)}_{\mathrm{h}}$ correspond to ${(100)}_{\mathrm{FCR}}$, ${(010)}_{\mathrm{FCR}}$, ${\left(001\right)}_{\mathrm{FCR}}$, respectively.

For EBSD analysis, the Cu_6_Sn_5_ phase is treated here as the high-temperature hexagonal *η* phase, and the low temperature monoclinic *η*${\prime}$ phase is considered later when discussing orientation relationships (Table 1). Attempts to index EBSD patterns from the Cu_6_Sn_5_ phase as monoclinic *η*${\prime}$ with the Hough transform method resulted in highly unreliable indexing due to the strong hexagonal pseudo-symmetry of the monoclinic *η*${\prime}$ phase. This issue is discussed in reference [61]. Further research, ideally with TEM-based diffraction, is required to determine whether the Cu6Sn5 phase in this work is hexagonal *η* or monoclinic *η*${\prime}$. The transformation from hexagonal (*η*) to monoclinic (*η*${\prime}$) axes can be calculated using [60]:

$$\left( {\begin{array}{*{20}c} {a_{{\mathrm{m}}} } \\ {b_{{\mathrm{m}}} } \\ {c_{{\mathrm{m}}} } \\ \end{array} } \right) = \left( {\begin{array}{*{20}c} 1 & 0 & 2 \\ { - 1} & { - 2} & 0 \\ 2 & 0 & { - 1} \\ \end{array} } \right)\left( {\begin{array}{*{20}c} {a_{{\mathrm{h}}} } \\ {b_{{\mathrm{h}}} } \\ {c_{{\mathrm{h}}} } \\ \end{array} } \right)$$

With this transformation matrix, the plane ${\left(2\bar{1 }\bar{1 }0\right)}_{\eta }$ corresponds to ${\left(102\right)}_{\eta {\prime}}$, and the directions ${[0\bar{1 }10]}_{\eta }$ and ${[0001]}_{\eta }$ correspond to ${\left[010\right]}_{\eta {\prime}}$ and ${[20\bar{1 }]}_{\eta {\prime}}$ respectively.

To measure the area fraction of surface-precipitated (Bi) particles, for each joint, one to five BSE images were collected at 5000 × magnification. ImageJ software was then used to threshold and binarise the (Bi) particles, before the Particle Analysis function was applied to measure the total area of (Bi). The area fraction of (Bi) was obtained by dividing the total area of (Bi) by the area of the image, and the mean (Bi) area fraction of each joint was obtained by averaging the (Bi) area fractions of all measured images.

## Results and discussion

### Effects of location and thermal history on (Bi) particles

**Figure 1:**
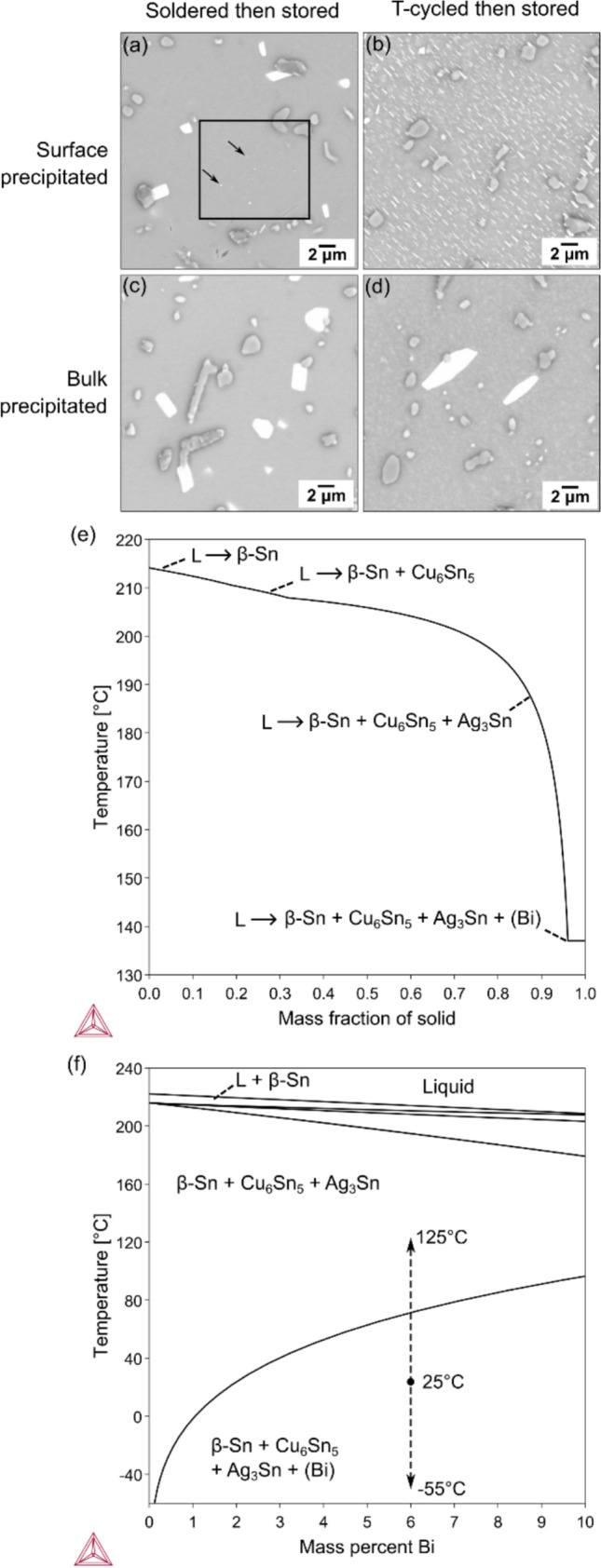
Dependence of (Bi) particle features on their location in solder joints and their thermal history. **a**–**d** BSE-SEM images where (Bi) particles are bright. The left column ((**a**) and (**c**)) were soldered-then-stored. The right column ((**b**) and (**d**)) were thermally-cycled-then-stored. The top row ((**a**) and (**b**)) highlight regions with surface precipitation, i.e. in (**a**), the small particles in the black box; in (**b**), all (Bi) particles. The bottom row ((**c**) and (**d**)) are regions with bulk-precipitated (Bi) that did not change after polishing. **e, f** Calculations in Thermo-Calc with the NIST solder database [67]. **e** Solidification reactions in Sn-2.25Ag-0.5Cu-6Bi from the Scheil model. **f** The (97.25-x)Sn-2.25Ag-0.5Cu-xBi isopleth with the temperature cycling range indicated at 6 wt. % Bi. In **e**, **f** the *α*-Sn phase and the polymorphs of Cu_6_Sn_5_ were not considered in the calculations.

In this work, the features of (Bi) particles depended on their location in the solder joints (in the bulk solder or on the free surface) and their thermal history (soldered-then-stored or thermally-cycled-then-stored). The key features are summarised in a 2 × 2 matrix in Table 2 and an example micrograph is given from each condition in Fig. 1a–d Note here that ‘surface precipitation’ refers to (Bi) precipitates that emerged gradually with time over days and weeks after polishing. ‘bulk precipitation’ refers to (Bi) precipitates that were relatively large shortly after polishing and did not develop significantly with time after polishing and, therefore, are assumed to have precipitated in the bulk before polishing. For example, in Fig. 1a, a few small surface-precipitated (Bi) are present in the black box that changed in size significantly in the days after polishing, whereas pre-existing bulk-precipitated (Bi) that did not change significantly in the days after polishing are present outside the black box.

**Table 2:** A summary of the features of (Bi) particles at different locations (in the bulk solder or on the free surface) with different thermal histories (soldered + 23 °C-aged or thermally cycled + 23 °C-aged)

	Soldered + stored at 23 °C for ~ 7 years	Soldered + -55/125 °C thermally cycled for 4876 cycles + stored at 23 °C for ~ 6 years
Surface-precipitated (Bi) after polishing	• Negligible surface precipitation • Very fine precipitates (< 500 nm) • Occasionally present for the first few days, then re-dissolved back into *β*-Sn • Area fraction of (Bi) negligible (< 0.001)	• Fine plates (< 2 μm) • Copious and uniform precipitation within *β*-Sn, similar to [53] • Most only share interfaces with *β*-Sn • ~ 80% have OR with *β*-Sn, and OR1 predominates • No OR with Cu_6_Sn_5_ and Ag_3_Sn • Area fraction of (Bi) between 0.01 and 0.15 (Fig. 9a)
Bulk-precipitated (Bi) existing before polishing	• Small (1 ~ 10 µm), blocky or plate-like • Distributed near eutectic regions • Often share interfaces with IMC particles • ~ 55% have an OR with *β*-Sn, where OR2 is more than OR1 • ~ 7% have OR with Cu_6_Sn_5_ • No OR with Ag_3_Sn • Area fraction of (Bi) between 0.005 and 0.02	• Large (3 ~ 20 μm), blocky or plate-like • Often share interfaces with IMC particles • ~ 65% have an OR with *β*-Sn, where OR2 is more than OR1 • No OR with Cu_6_Sn_5_ and Ag_3_Sn • Area fraction of (Bi) between 0.01 and 0.03

In the soldered-then-stored joints (left side of Table 2 and Fig. 1a–d), the bulk (Bi) particles are a combination of (i) those that initially formed by a eutectic reaction during non-equilibrium solidification, as shown in the Scheil path in Fig. 1e, and then coarsened; and (ii) those that formed by solid-state precipitation and coarsening from the supersaturated *β*-Sn matrix during cooling and room-temperature storage, as can be seen in the isopleth in Fig. 1f. The lack of surface precipitation indicates that the Bi concentration in the *β*-Sn matrix approached equilibrium during storage for this thermal history. Compared with past work, Belyakov et al. [51] and Wu et al. [41] polished joints shortly after soldering and observed copious surface precipitation and coarsening whereas, in the current study, we polished joints > 7 years after soldering and observed almost no subsequent surface precipitation.

In the thermally cycled-then-stored joints (right side of Table 2 and Fig. 1a–d), the (Bi) phase dissolved in the *β*-Sn matrix in the hot part of the thermal cycles and the bulk (Bi) particles precipitated from the supersaturated *β*-Sn matrix on cooling and coarsened during the following six years of room-temperature storage (see the isopleth in Fig. 1f). After polishing, (Bi) particles precipitated copiously and uniformly from *β*-Sn at the new surface, similar to our previous time-lapse study [53], indicating that the *β*-Sn was still supersaturated in Bi for this thermal history.

These different ‘types’ of (Bi) particles behaved differently in terms of their (i) distribution and morphology, (ii) orientation relationship (ORs) with *β*-Sn (OR1 or OR2), and (iii) OR with IMC phases (OR with Cu_6_Sn_5_ or not), as summarised in Table 2. The key findings are that (Bi) particles precipitating on the surface tended to have OR1 with *β*-Sn after coarsening, while (Bi) particles in the bulk exhibited both OR1 and OR2 with *β*-Sn, with a preference for OR2. Surface-precipitated (Bi) mostly shared interfaces with *β*-Sn, whereas more than 60% of bulk-precipitated (Bi) shared interfaces with both *β*-Sn and IMC particles, as can be seen in Fig. 1a–d, and are quantified in detail in Section “Orientation relationships between (Bi) and IMCs”. In most cases, there was no OR between the (Bi) and IMCs but, in soldered-then-stored joints, ~ 7% of bulk-precipitated (Bi) particles had an OR with a Cu_6_Sn_5_ particle. No ORs between (Bi) and Ag_3_Sn were detected in this work. These features are presented and discussed in detail in the remainder of this paper.

No significant differences in (Bi) precipitates were found between the joints of the 84CTBGA and 192CABGA packages, as can be seen from SI-Fig. 2 in the Supplementary Information; the results in Table 2 and Fig. 1 are valid for both package types. This is mostly because both packages underwent the same thermal history involving long-term ageing and the solder balls of both packages were of the same composition. Therefore, for the remainder of this paper, the results from these two package types are not separated and examples are given from both.

A further important result in Fig. 1a–d and Table 2 that is significant for the long-term reliability of Sn–Ag–Cu–Bi solder joints is that, after the thermal histories studied here, the bulk-precipitated (Bi) particles are large (> 1 μm) and widely spaced (> 9 μm) relative to the precipitation strengthening mechanisms of dislocation cutting and bowing. Thus, using this Sn–Ag–Cu–Bi solder alloy in electronic applications that involve long-term storage at room temperature would result in over-aged joints with an expected loss of strength.

### Orientation relationships between (Bi) and β-Sn

#### Orientation relationship 1

**Figure 2:**
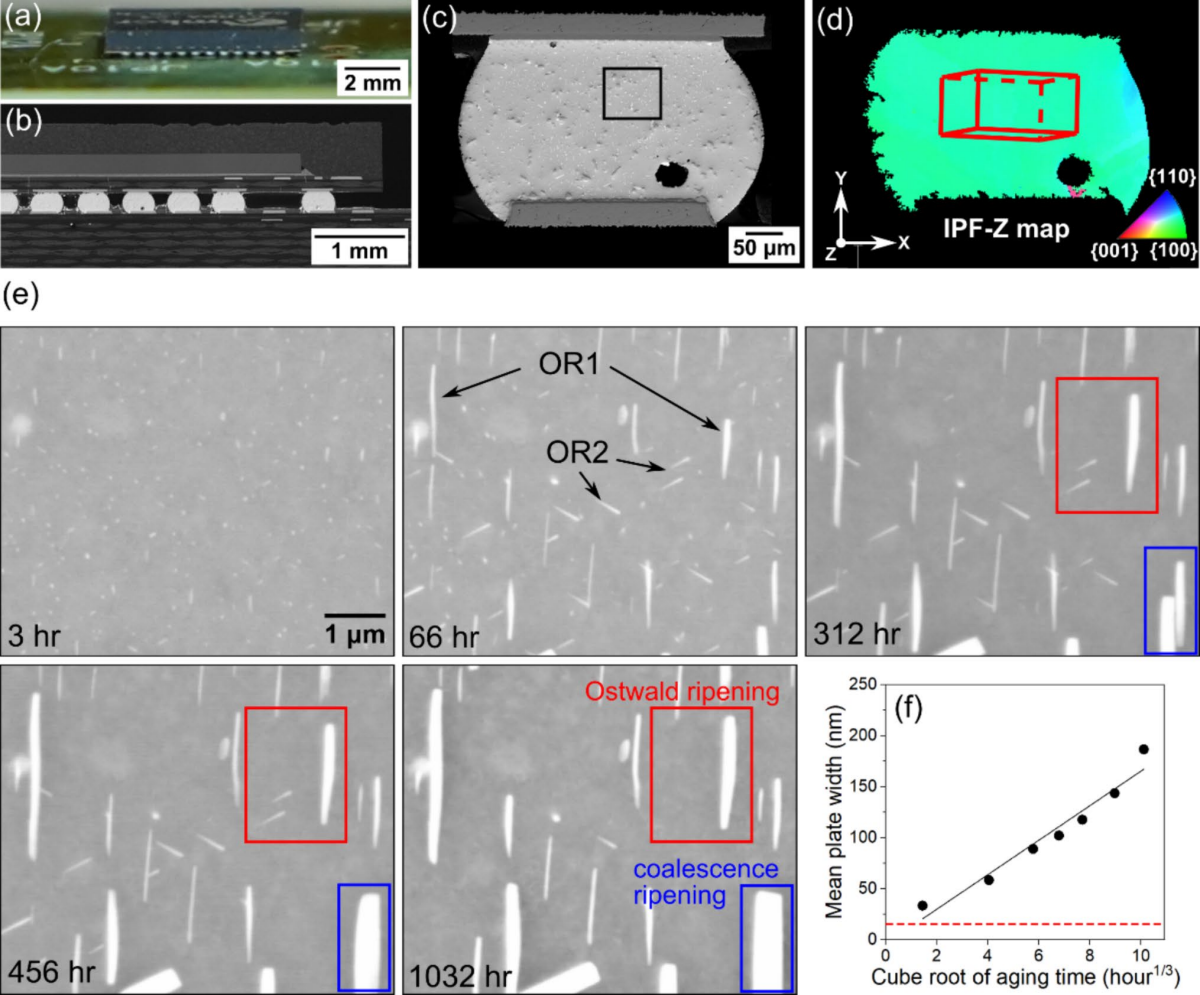
Competition between *β*-Sn/(Bi) OR1 and OR2 on a free surface after polishing. **a** Photograph and **b** micrograph of 84CTBGA package soldered to a printed circuit board. **c** BSE image showing the location of the area in (**e**). **d** EBSD orientation map of the *β*-Sn phase (IPF-Z) superimposed with a *β*-Sn unit-cell wireframe indicating the orientation of this single-grained joint. **e** Time-lapse BSE images showing the evolution of (Bi) particles at five times. This example is from surface-precipitated (Bi) in a thermally-cycled-then-stored 84CTBGA joint. **f** Mean (Bi) plate width versus cube root of ageing time. The data was collected from a larger area containing the area of (**e**), and the red dashed line represents the pixel size of the BSE images.

**Figure 3:**
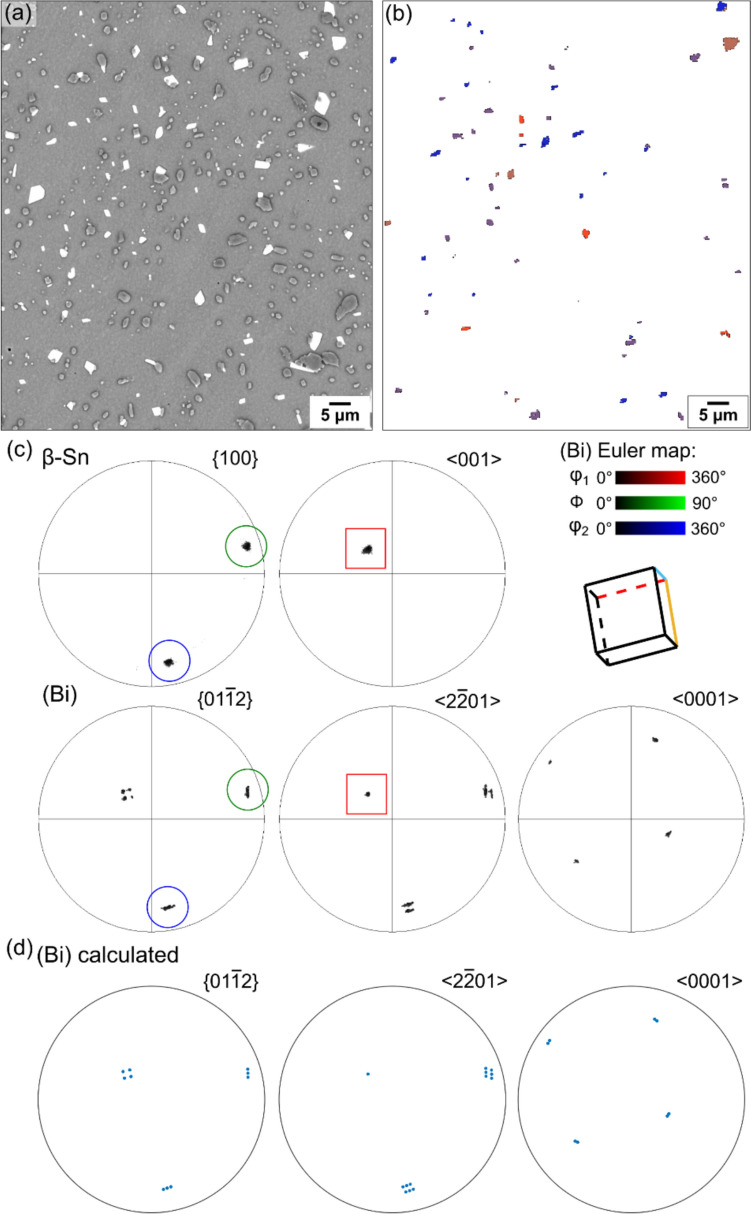
Orientation variants of OR1. **a** BSE image. **b** EBSD Euler map of the (Bi) particles with OR1. **c** *β*-Sn and (Bi) pole figures for (Bi) particles with OR1. The inset *β*-Sn unit cell was plotted from the measured Euler angles. **d** 8 theoretical (Bi) variants calculated according to OR1. This example is from surface-precipitated (Bi) in a thermally-cycled-then-stored 84CTBGA joint.

**Figure 4:**
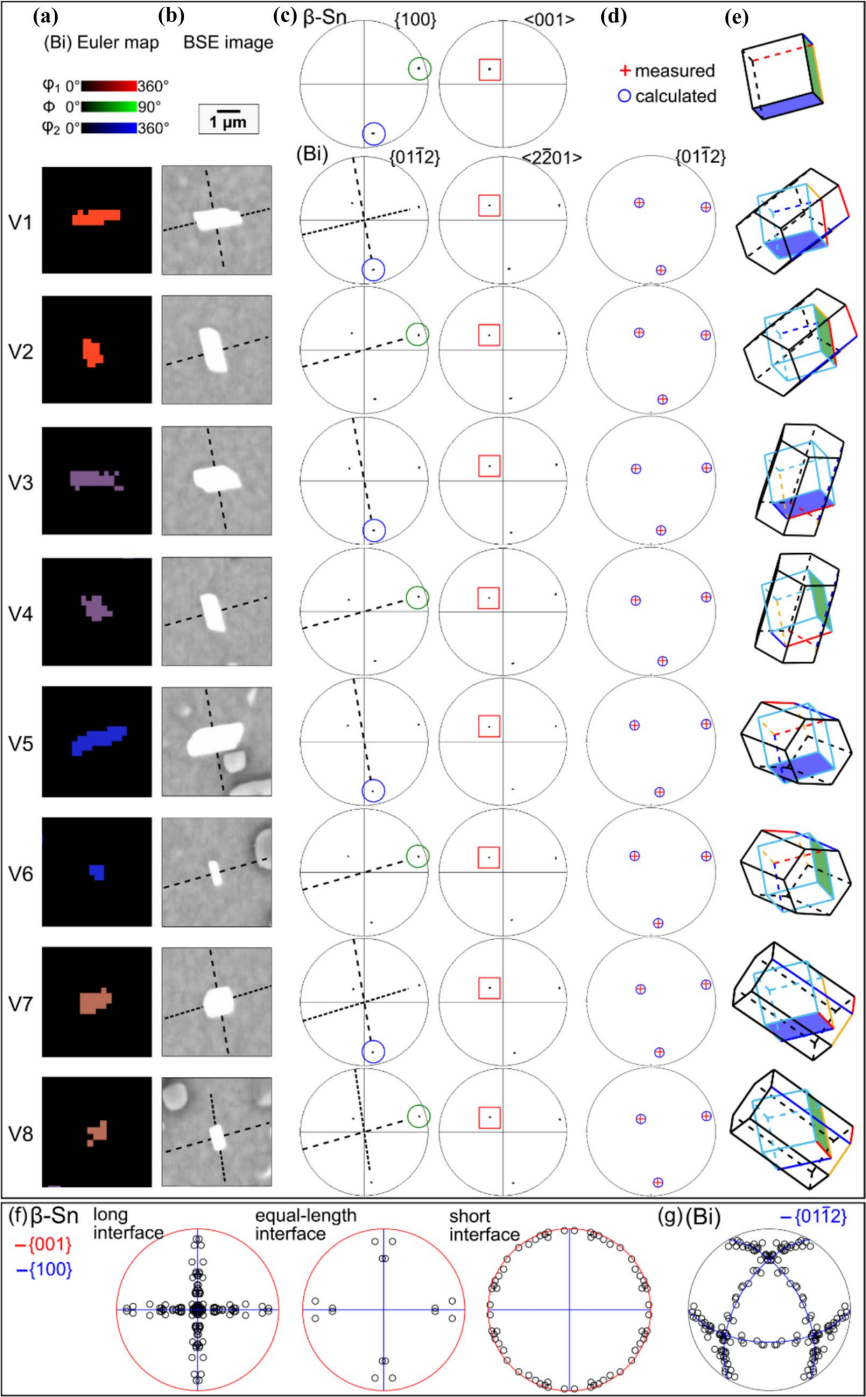
Individual (Bi) particles with the 8 orientation variants of OR1 extracted from Fig. 3. **a** EBSD Euler maps and **b** BSE images. **c** EBSD pole figures of (Bi) and *β*-Sn with overlapped planes and axes highlighted. **d** ${\{01\bar{1}2\} }_{(\mathrm{Bi})}$ pole figures of calculated OR1 orientation variants (red cross) overlayed with experimentally measured OR1 orientation variants of (Bi) particles (blue circle). **e** Unit-cell wireframes of *β*-Sn and the (Bi) hexagonal unit cells, and (Bi) face-centred rhombohedral unit cells, with parallel ${{\{100\}}_{\mathrm{Sn}}/\{01\bar{1}2\} }_{(\mathrm{Bi})}$ planes coloured. **f**, **g** Inverse pole figures (IPF) of *β*-Sn and (Bi) summarising the lattice directions lying in (Bi)/*β*-Sn interface planes in BSE images of (Bi) particles with OR1. For the IPF of *β*-Sn, the data are separately plotted for long interfaces, equal-length interfaces, and short interfaces. The blue and red lines in (**f**) mark the traces of $\{100\}$ and $\{001\}$ planes of *β*-Sn, respectively. The blue lines in (**g**) mark the traces of $\left\{ {01\bar{1}2} \right\}$ planes of (Bi).

**Figure 5:**
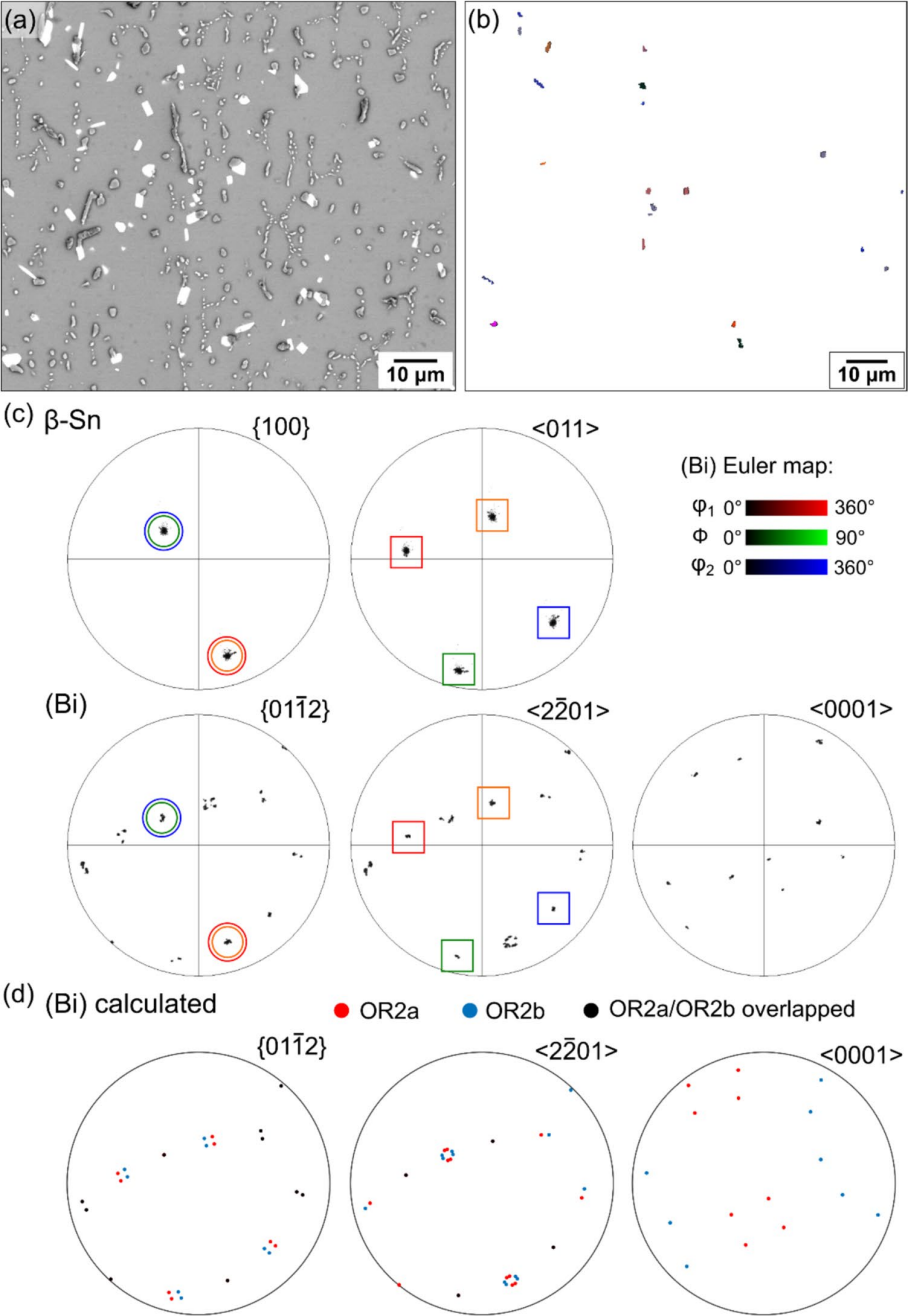
Orientation variants of OR2. **a**–**c** An area with (Bi) particles showing OR2a and OR2b orientation variants. **a** BSE image of an area. **b** EBSD Euler map of the (Bi) particles showing OR2a and OR2b. **c** Selected *β*-Sn and (Bi) pole figures for (Bi) particles with OR2a and OR2b. **d** 16 theoretical (Bi) variants calculated according to OR2a and OR2b. Note that only 10 (Bi) orientation variants were measured in (**b**, **c**). This example is from bulk-precipitated (Bi) in a soldered-then-stored 192CABGA joint.

**Figure 6:**
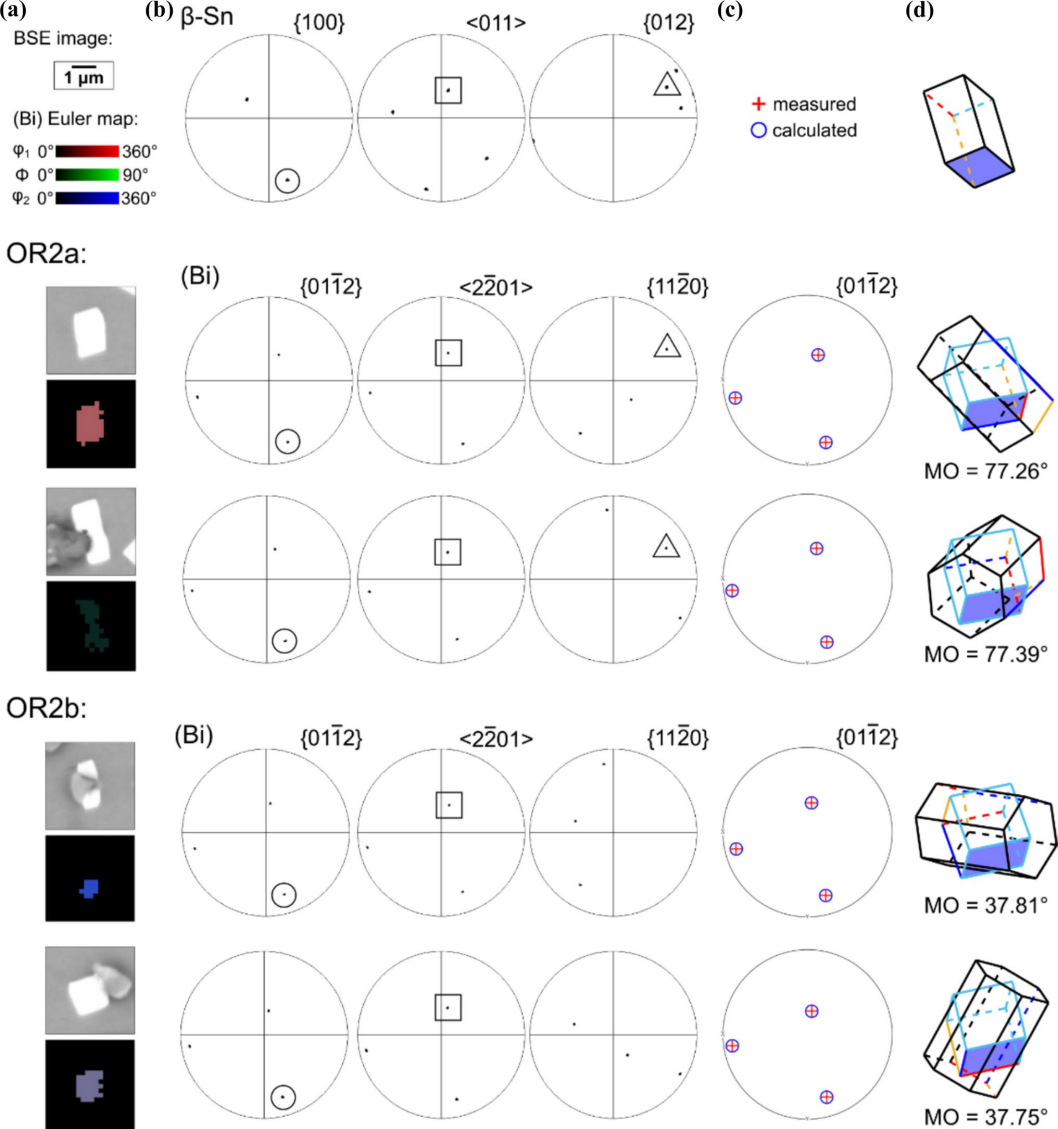
Distinguishing between OR2a and OR2b in Fig. 5 in four (Bi) particles, all with their ${\{01\bar{1}2\} }_{(\mathrm{Bi})}$ and ${\langle 2\bar{2}01\rangle }_{(\mathrm{Bi})}$ parallel with the same member of ${\{100\}}_{\beta \mathrm{Sn}}$ and ${\langle 011\rangle }_{\beta \mathrm{Sn}}$. **a** BSE images and EBSD Euler maps. **b** Pole figures highlighting parallel planes and directions. **c** $\left\{ {01\bar{1}2} \right\}$ pole figures of calculated OR2 variants (red crosses) overlayed with experimentally measured OR2 variants of (Bi) (blue circles). **d** Wireframes of the *β*-Sn tetragonal unit cell, (Bi) hexagonal unit cells, and (Bi) FCR unit cells, with the overlapped ${\{100\}}_{\beta \mathrm{Sn}}/{\{01\bar{1}2\} }_{(\mathrm{Bi})}$ planes shaded in blue. Note that OR2a has ${\{012\}}_{\beta \mathrm{Sn}}/{\{11\bar{2}0\} }_{(\mathrm{Bi})}$ whereas OR2b does not.

**Figure 7:**
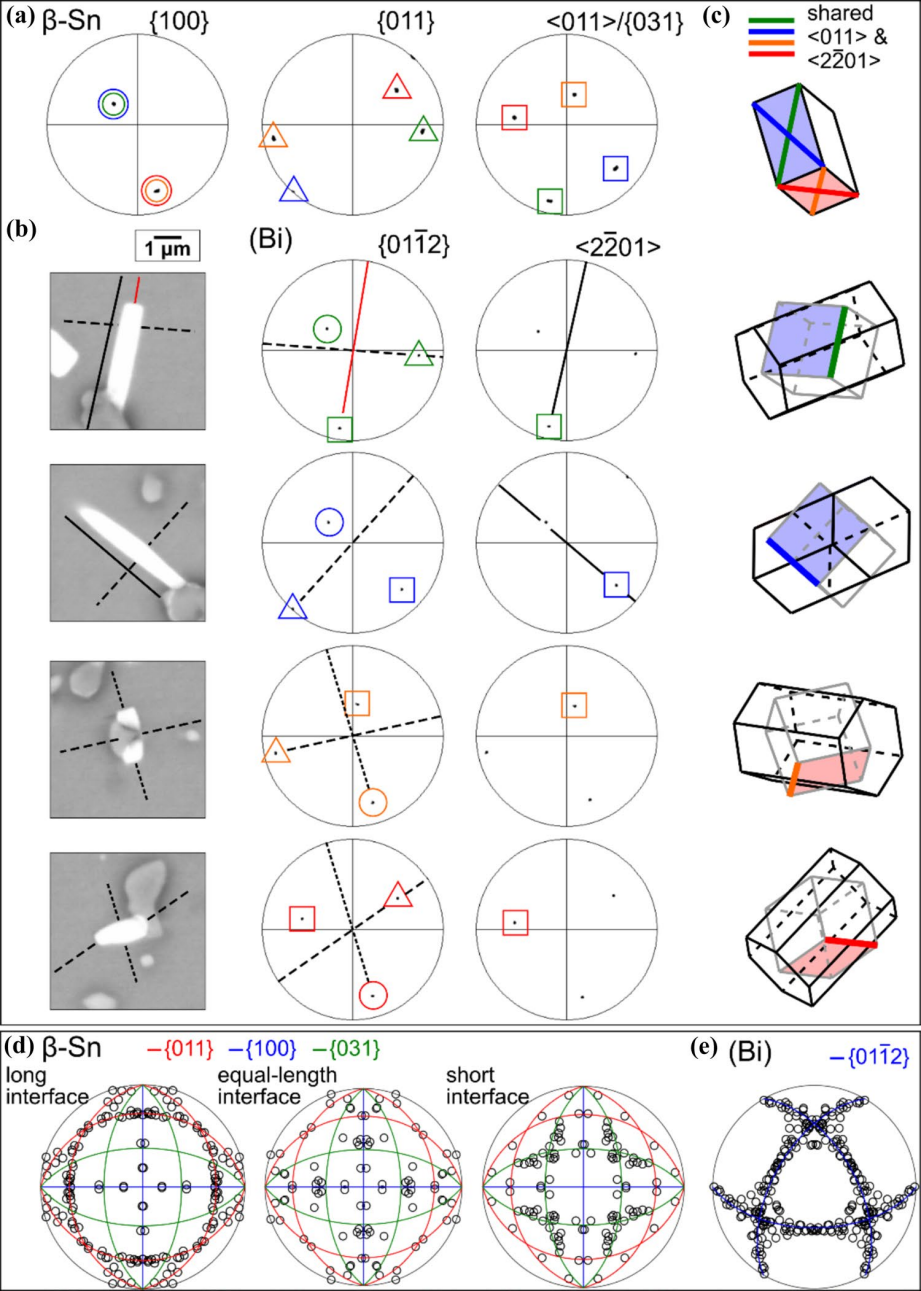
Interfaces in OR2. **a**–**c**: Four (Bi) particles with different OR2b variant orientations in the area shown in Fig. 5. **a** Pole figures of *β*-Sn matrix and the (Bi) particles. **b** BSE images of the (Bi) particles. **c** Wireframe unit cells of *β*-Sn, (Bi) hexagonal unit cells, and (Bi) FCR unit cells, with the shared ${\langle 011\rangle }_{\beta \mathrm{Sn}}$ and ${\langle 2\bar{2}01\rangle }_{(\mathrm{Bi})}$ axes highlighted in four colours. Note there are eight variants for OR2b but only four are shown here. **d**, **e** Inverse pole figure (IPF) analysis of (Bi)/*β*-Sn interfaces with OR2a and OR2b. IPFs of *β*-Sn and (Bi) summarise the lattice directions lying in (Bi)/*β*-Sn interface planes measured from BSE images. For the IPF of *β*-Sn, the data are separately plotted for long interfaces, equal-length interfaces, and short interfaces.

Figure 2 overviews the general features of precipitation and coarsening of (Bi) particles on the free surface of thermally cycled Sn-2.25Ag-0.5Cu-6Bi joints in the 84CTBGA package. Figure 2a displays the whole 84CTBGA package and Fig. 2b is a cross-sectional view of the joints in one row. Figure 2c shows the Sn-2.25Ag-0.5Cu-6Bi joint in this example, which contains a single *β*-Sn grain with its c-axis oriented near-vertically (Fig. 2d). As highlighted with arrows in the BSE image at 66 h (Fig. 2e), the (Bi) particles are initially aligned along three main directions. The vertical plates correspond to OR1, and the two diagonal plate orientations both have OR2 with the *β*-Sn matrix. These ORs were confirmed by EBSD, similar to Figs. 3, 4, 5, 6, 7 later in this paper, and TEM analysis of this OR is available elsewhere [68]. The time-lapse images in Fig. 2e show that, during the 1032 h’ ageing at room temperature, the surface (Bi) particles with OR1 grew at the expense of those with OR2. This observation is in accordance with past work on the surface precipitation of (Bi) [51].

In a recent time-lapse study on similar solder joints [53] we showed that, during surface coarsening, the (Bi) particle size scales with the cube root of ageing time at room temperature, consistent with both the kinetics of Ostwald ripening within Lifshitz–Slyozov–Wagner (LSW) theory [69, 70] and with coalescence ripening within Takajo theory [71]. The time-lapse observations confirmed that both Ostwald ripening and coalescence ripening occur concurrently. Similar behaviour is shown in Fig. 2, which demonstrates that surface coarsening of (Bi) particles is governed by two mechanisms: (i) the growth of large (Bi) particles at the expense of smaller (Bi) particles through the diffusion of Bi in *β*-Sn as shown in the red box in Fig. 2e , and (ii) the merging of two (Bi) particles which forms anomalously large (Bi) particles as shown in the blue box in Fig. 2e, f further confirms that the variation in precipitate size approximately scales with the cube root of ageing time on the surface of this joint.

To study OR1, EBSD analysis was applied on an area containing a single *β*-Sn grain and multiple surface-precipitated (Bi) particles in a thermally cycled Sn-2.25Ag-0.5Cu-6Bi 84CTBGA joint, as displayed in Fig. 3. Note that the (Bi) precipitates in Fig. 3 have a different cross-sectional morphology to those in Fig. 2e because the *β*-Sn grain orientation is different. The (Bi) precipitates are elongated along the c-direction of *β*-Sn and the c-axis is almost in the plane of the page in Fig. 2e and nearly perpendicular to the page in Fig. 3.

The EBSD Euler map in Fig. 3b shows only the (Bi) particles with OR1, and the EBSD pole figures (Fig. 3c) summarise the orientations of these OR1 (Bi) particles and the *β*-Sn matrix, with the overlapped ${\left\{100\right\}}_{\beta \mathrm{Sn}}{\parallel \left\{01\bar{1}2\right\} }_{\left(\mathrm{Bi}\right)}$ planes and ${\langle 001\rangle }_{\beta \mathrm{Sn}}{\parallel \langle 2\bar{2}01\rangle }_{\left(\mathrm{Bi}\right)}$ axes highlighted with blue circles and red squares, respectively. In the Euler map (Fig. 3b), multiple colours can be seen, indicating multiple orientation variants of (Bi) with OR1. The (Bi) pole figures (Fig. 3c) show separate but closely spaced spots, e.g. the four spots near the centre of the ${\left\{01\bar{1}2\right\} }_{\left(\mathrm{Bi}\right)}$ pole figure. To verify the orientation variants, the MTEX toolbox was used to calculate the (Bi) orientations produced from the average *β*-Sn orientation in the area in Fig. 3a assuming OR1. We used point group symmetry 4/mmm for *β*-Sn and -3 m for (Bi), defined the OR by the parallel plane and direction written in OR-1 and calculated all symmetrically equivalent (Bi) orientations related to the parent *β*-Sn orientation. Eight orientation variants were calculated and are plotted as pole figures in Fig. 3d, showing a good match with the measured (Bi) orientation variants in Fig. 3c (average misorientation angle between the theoretical OR and the measured OR of ~ 1.12°).

Figure 4 demonstrates the eight orientation variants (V1 to V8) of OR1, where eight (Bi) particles were selected from the same area as Fig. 3, each representing an orientation variant. The eight variants were verified by matching the pole figures from EBSD with the calculated pole figures (Fig. 4d). In the Euler maps (Fig. 4a), the (Bi) particles have four distinct colours. Figiure 4e displays unit-cell wireframes plotted from the measured Euler angles to differentiate the orientation variants, using both the hexagonal and FCR unit cells of (Bi). The hexagonal unit cells are oriented in four groups corresponding to the four colours of (Bi) in the Euler maps (Fig. 4a), where each group contains two (Bi) orientations differentiated by whether the ${\left\{01\bar{1}2\right\} }_{(\mathrm{Bi})}$ is parallel with ${(100)}_{\beta \mathrm{Sn}}$ or ${(010)}_{\beta \mathrm{Sn}}$, as shown with blue and green colour in Fig. 4e. Note that parallel blue planes are inequivalent to parallel green planes in Fig. 4e because the angle between adjacent ${\left\{01\bar{1}2\right\} }_{(\mathrm{Bi})}$ is ~ 87.5° whereas the angle between ${\left\{100\right\}}_{\beta \mathrm{Sn}}$ is 90° and, for each orientation variant, only one ${\left\{01\bar{1}2\right\} }_{(\mathrm{Bi})}$ plane can overlap with either ${\left(100\right)}_{\beta \mathrm{Sn}}$ or ${\left(010\right)}_{\beta \mathrm{Sn}}$ for unstrained crystals. Examining the FCR unit cells in (*e*), note that all (Bi) variants have a similar pseudo-cubic orientation.

Comparing the dashed lines indicating interface plane normals in the BSE images (Fig. 4b) with the perpendicular bisectors in the ${\left\{01\bar{1}2\right\} }_{(\mathrm{Bi})}$ pole figures (Fig. 4c), we see that the largest interface of each (Bi) particle generally corresponds to the overlapped ${\left\{100\right\}}_{\beta \mathrm{Sn}}{\langle 001\rangle }_{\beta \mathrm{Sn}}{\parallel \left\{01\bar{1}2\right\} }_{\left(\mathrm{Bi}\right)}{\langle 2\bar{2}01\rangle }_{(\mathrm{Bi})}$ interface. The shorter interfaces are either curved or contain segments corresponding to the non-overlapped ${\left\{100\right\}}_{\beta \mathrm{Sn}}$/${\left\{01\bar{1}2\right\} }_{\left(\mathrm{Bi}\right)}$ planes, which cannot be parallel due to the 87.5° versus 90° angular mismatch. This is particularly clear for particle V1, V7, and V8, where the finer dashed lines, pointing towards the non-overlapped ${\left\{100\right\}}_{\beta \mathrm{Sn}}$/${\left\{01\bar{1}2\right\} }_{\left(\mathrm{Bi}\right)}$ planes in the (Bi) pole figures (Fig. 4c), are also perpendicular to the shorter interface in the SEM images (Fig. 4b).

To examine the preference of *β*-Sn/(Bi) interfaces in OR1, we measured the lattice directions lying in straight (Bi)/*β*-Sn interfaces in the cross sections (BSE images) of 20 (Bi) particles with OR1. The measured lattice directions are summarised in inverse pole figures (IPF) of *β*-Sn and (Bi) in Fig. 4f, g. For the IPF of (Bi) in Fig. 4g, all measured data are plotted and the traces of $\left\{ {01\bar{1}2} \right\}$ planes are marked by blue lines. Nearly all measured directions lie on the ${\{01\bar{1}2\} }_{(\mathrm{Bi})}$ traces, indicating that (Bi) precipitates generally had $\left\{ {01\bar{1}2} \right\}$ interfaces in OR1 in this work.

For the IPF of *β*-Sn (Fig. 4f), the data are categorised into: (i) long interfaces, where one interface is at least twice as long as the others in the cross section of a (Bi) particle; (ii) short interfaces, where one interface is less than half the length of the others in the sectioned (Bi) particle; and (iii) equal-length interfaces, where the longest interface is no more than twice the length of the shortest interface (e.g. V7 in Fig. 4). The traces of $\{100\}$ and $\{001\}$ planes are marked by blue and red lines, respectively. Comparing the three IPFs (Fig. 4f), we see that all the directions lying in long interfaces lie close to the traces of ${\{100\}}_{\beta Sn}$, while all the directions along short interfaces lie close to the ${\{001\}}_{\beta Sn}$ traces. These measurements demonstrate that, in OR1, (Bi) particles mainly have two habit planes: ${\{100\}}_{\beta \mathrm{Sn}}\parallel {\{01\bar{1}2\} }_{(\mathrm{Bi})}$ as the major interface and ${\{001\}}_{\beta \mathrm{Sn}}\parallel {\{01\bar{1}2\} }_{(\mathrm{Bi})}$ as the minor interface although, again, the latter cannot be exactly parallel due to the 87.5° versus 90° angular mismatch.

#### Orientation relationship 2

To investigate OR2, Fig. 5 shows a selected area containing a single *β*-Sn grain and multiple bulk-precipitated (Bi) particles. This example is from a Sn-2.25Ag-0.5Cu-6Bi 192CABGA joint that had been room temperature aged after soldering. The EBSD Euler map (Fig. 5b) displays only the (Bi) particles sharing OR2 with the *β*-Sn matrix, and the EBSD pole figures (Fig. 5c) summarise the orientations of these OR2 (Bi) particles and the *β*-Sn matrix, with the overlapped ${\left\{100\right\}}_{\beta \mathrm{Sn}}{\parallel \left\{01\bar{1}2\right\} }_{\left(\mathrm{Bi}\right)}$ planes and ${\langle 011\rangle }_{\beta \mathrm{Sn}}{\parallel \langle 2\bar{2}01\rangle }_{\left(\mathrm{Bi}\right)}$ axes highlighted. Similar to OR1 (Fig. 4), the Euler map shows multiple colours of (Bi) (Fig. 5b), and the (Bi) pole figures show clusters of separated spots (Fig. 5c), indicating multiple orientation variants for OR2. Calculations with MTEX revealed 16 orientation variants for (Bi) particles with OR2 (Fig. 5d), but only 10 of them were measured in this area, as indicated by the 10 spots in the ${\langle 0001\rangle }_{(\mathrm{Bi})}$ EBSD pole figure (Fig. 5c) compared with the 16 calculated spots in ${\langle 0001\rangle }_{(\mathrm{Bi})}$ in Fig. 5d. The measured 10 variants (Fig. 5c) closely match with 10 out of the 16 calculated variants (Fig. 5d), with an average misorientation angle between the theoretical OR and the measured OR of ~ 1.18°.

Note in Fig. 5d that the orientation variants are separated into OR2a (red) and OR2b (blue) which are two different orientation relationships. This is demonstrated in Fig 6 where OR2a has ${\{012\}}_{\beta \mathrm{Sn}}$ overlapping with ${\{11\bar{2}0\} }_{(\mathrm{Bi})}$, whereas OR2b does not (Fig. 6b). The misorientation (MO) angle between (Bi) and *β*-Sn is ~ 77.3° for OR2a, compared to ~ 37.8° for OR2b (Fig. 6d). These are two closely related ORs that can be written:

OR2a $$\left\{ {100} \right\}_{{\beta {\mathrm{Sn}}}} \parallel \left\{ {01\bar{1}2} \right\}_{{\left( {{\mathrm{Bi}}} \right)}} {\mathrm{and}} \left\langle {011} \right\rangle_{{\beta {\mathrm{Sn}}}}  \parallel \left\langle {2\bar{2}01} \right\rangle_{{\left( {{\mathrm{Bi}}} \right)}}$$

OR2b $$\left\{ {100} \right\}_{{\beta {\mathrm{Sn}}}} \parallel \left\{ {\bar{1}012} \right\}_{{\left( {{\mathrm{Bi}}} \right)}} {\mathrm{and}}\left\langle {011} \right\rangle _{{\beta {\mathrm{Sn}}}} \parallel \left\langle {2\bar{2}01} \right\rangle _{{\left( {{\mathrm{Bi}}} \right)}}$$

The 16 variants of OR2 calculated in Fig. 5d are made up of eight variants of OR2a and eight variants of OR2b.

To determine the preferred interfaces in OR2, we measured the lattice directions along straight (Bi)/*β*-Sn interfaces in the cross sections (BSE images) of 28 (Bi) particles with OR2. The measured lattice directions are summarised in the inverse pole figures (IPFs) of *β*-Sn (Fig. 7d) and (Bi) (Fig. 7e). Note, the data here for OR2a and OR2b are combined, as both ORs display similar distributions in the IPFs. For *β*-Sn (Fig. 7d), the IPF is separately plotted for long interfaces (left), equal-length interfaces (middle), and short interfaces (right), using the same definition as in the *β*-Sn IPFs for OR1. The traces of $\{100\}$, $\{011\}$, and $\{031\}$ planes are marked by blue, red, and green lines, respectively. In the *β*-Sn IPFs (Fig. 7d), we see that long interfaces have most of their directions lying in ${\{011\}}_{\beta \mathrm{Sn}}$ planes, whereas short interfaces have most of their directions lying close to ${\{031\}}_{\beta \mathrm{Sn}}$ planes. Equal-length interfaces show no clear preference, but they have slightly more directions lying in ${\{100\}}_{\beta \mathrm{Sn}}$ planes than in ${\{011\}}_{\beta \mathrm{Sn}}$ and ${\{031\}}_{\beta \mathrm{Sn}}$ planes. For the IPF of (Bi) (Fig. 7e), nearly all measured directions in (Bi) interfaces are on the traces of ${\{01\bar{1}2\} }_{(\mathrm{Bi})}$ planes marked by blue lines. Thus, (Bi) particles usually have $\left\{ {01\bar{1}2} \right\}$ interfaces in OR2, the same as in OR1. The main habit planes were ${\{011\}}_{\beta \mathrm{Sn}}\parallel {\{01\bar{1}2\} }_{(\mathrm{Bi})}$, followed by ${\{100\}}_{\beta \mathrm{Sn}}\parallel {\{01\bar{1}2\} }_{(\mathrm{Bi})}$, while ${\{031\}}_{\beta \mathrm{Sn}}\parallel {\{01\bar{1}2\} }_{(\mathrm{Bi})}$ tends to form as the smallest interface. As with OR1, when the first pair of planes are parallel, the latter two pairs cannot be perfectly parallel due to the small angular mismatch between *β*-Sn and (Bi). In short, the strong preference for lattice vectors in long interfaces to lie on the trace of ${\{011\}}_{\beta \mathrm{Sn}}$ in the IPF in Fig. 7d indicates a preference for long interfaces to be ${\left\{011\right\}}_{\beta \mathrm{Sn}}{\parallel \left\{01\bar{1}2\right\} }_{\left(Bi\right)}$ in OR2.

The preference for long interfaces to be ${\left\{011\right\}}_{\beta \mathrm{Sn}}{\parallel \left\{01\bar{1}2\right\} }_{\left(\mathrm{Bi}\right)}$ in OR2, as manifested by the accumulation along the trace of ${\left\{011\right\}}_{\beta \mathrm{Sn}}$ in the IPF of long interfaces (Fig. 7d), can also be seen in the examples in Fig. 7b by comparing the dashed lines in the BSE images with the dashed perpendicular bisectors in the (Bi) pole figures. The solid lines on the first two (Bi) particles in Fig. 7b also highlight that the (Bi) particles tended to be elongated along the ${\langle 011\rangle }_{\beta \mathrm{Sn}}{\parallel \langle 2\bar{2}01\rangle }_{\left(\mathrm{Bi}\right)}$ axes. For the two lower (Bi) particles in Fig. 7b, we also see a ${\left\{100\right\}}_{\beta \mathrm{Sn}}{\langle 011\rangle }_{\beta \mathrm{Sn}}{\parallel \left\{01\bar{1}2\right\} }_{\left(\mathrm{Bi}\right)}{\langle 2\bar{2}01\rangle }_{(\mathrm{Bi})}$ interface, as indicated by the finer dashed lines. In contrast, ${\left\{031\right\}}_{\beta \mathrm{Sn}}{\langle 100\rangle }_{\beta \mathrm{Sn}}{\parallel \left\{01\bar{1}2\right\} }_{\left(\mathrm{Bi}\right)}{\langle 2\bar{2}01\rangle }_{(\mathrm{Bi})}$ only appears as a short interface at the top of the upper (Bi) particle in Fig. 7b , as indicated by the red line perpendicular to the short interface in the BSE image (Fig. 7b) which is colinear with the overlapped ${\left\{031\right\}}_{\beta \mathrm{Sn}}{\parallel \left\{01\bar{1}2\right\} }_{\left(\mathrm{Bi}\right)}$ plane normal (green square) in the (Bi) $\left\{ {01\bar{1}2} \right\}$ pole figure (Fig. 7a).

#### Competition between (Bi) precipitates with OR1 and OR2 to β-Sn

In Fig. 2 and reference [51], surface-precipitated (Bi) particles with OR1 outcompeted those with OR2 during ageing. Belyakov et al. [51] attributed this to the more coherent (Bi)/*β*-Sn interface. However, they only considered the coherency of one interface for each OR, while (Bi) precipitates are three-dimensional with multiple interface types. For example, for OR1, the main interfaces are the symmetrically equivalent ${(100)}_{\beta \mathrm{Sn}}\parallel {(01\bar{1 }2)}_{(\mathrm{Bi})}$ and ${(010)}_{\beta \mathrm{Sn}}\parallel {(10\bar{1 }\bar{2 })}_{(\mathrm{Bi})}$ and the minor interface is ${(001)}_{\beta \mathrm{Sn}}\parallel {(1\bar{1 }02)}_{(\mathrm{Bi})}$. In contrast, for OR2, the main interface is ${\left\{011\right\}}_{\beta \mathrm{Sn}}{\langle 01\bar{1}\rangle }_{\beta \mathrm{Sn}}{\parallel \left\{01\bar{1}2\right\} }_{\left(\mathrm{Bi}\right)}{\langle 2\bar{2}01\rangle }_{(\mathrm{Bi})}$ type with minor interfaces of ${\left\{100\right\}}_{\beta \mathrm{Sn}}{\langle 011\rangle }_{\beta \mathrm{Sn}}{\parallel \left\{01\bar{1}2\right\} }_{\left(\mathrm{Bi}\right)}{\langle 2\bar{2}01\rangle }_{(\mathrm{Bi})}$ and ${\left\{0\bar{3}1\right\} }_{\beta \mathrm{Sn}}{\langle 100\rangle }_{\beta \mathrm{Sn}}{\parallel \left\{10\bar{1 }\bar{2}\right\} }_{\left(\mathrm{Bi}\right)}{\langle \bar{2 }20\bar{1}\rangle }_{(\mathrm{Bi})}$. To consider the interfacial coherencies of OR1 and OR2, the planar disregistries of these interfaces for each OR were calculated using the method proposed by Bramfitt [72], and the results are listed in Table 3. Note, as ~ 1.2 at.% of Bi was dissolved in *β*-Sn in Sn-2.25Ag-0.5Cu-6Bi at 23 °C [67], the effect of dissolved Bi on the lattice spacings of *β*-Sn was considered. The *β*-Sn lattice spacings in Table 3 were modified from those in Table 1 according to the *β*-Sn lattice spacing – Bi concentration relationship reported by Lee and Raynor [73]. The procedure is demonstrated in SI-Fig.3.

**Table 3:** The results of the planar disregistry calculation for the *β*-Sn/(Bi) interfaces in OR1 (top), OR2a (middle), and the *β*-Sn/Cu_6_Sn_5_ interfaces in the *β*-Sn/Cu_6_Sn_5_ OR (bottom). d[uvw] is the mean inter-atomic distance of a lattice direction; θ is the angle between two directions; and *δ* is the planar disregistry of an interface

*β*-Sn/(Bi) OR1	(1 0 0)Sn//(0 1 −1 2)Bi			(0 1 0)Sn//(1 0 −1 −2)Bi			(0 0 1)Sn//(1 −1 0 2)Bi		
[uvw]Sn	[0 0 1]	[0 −1 2]	[0 −1 0]	[0 0 1]	[1 0 2]	[1 0 0]	[0 1 0]	[−1 1 0]	[−1 0 0]
[uvw]Bi	[2 −2 0 1]	[2 −1 −1 0]	[2 0 −2 −1]	[2 −2 0 1]	[1 −2 1 0]	[0 −2 2 −1]	[2 0 −2 −1]	[1 1 −2 0]	[0 2 −2 1]
d[uvw]Sn (Å)	3.188	4.321	2.917	3.188	4.321	2.917	2.917	4.125	2.917
d[uvw]Bi (Å)	3.286	4.546	3.286	3.286	4.546	3.286	3.286	4.546	3.286
*θ* (°)	0	3.779	2.464	0	3.779	2.464	0	1.232	2.464
*δ* (%)	6.5			6.5			10.6		

*β*-Sn/(Bi) OR2	(1 0 0)Sn//(−1 1 0 −2)Bi			(0 1 1)Sn//(0 1 −1 2)Bi			(0 −3 1)Sn//(1 0 −1 −2)Bi		
[uvw]Sn	[0 −1 1]	[0 −2 −1]	[0 −1 −3]	[0 −1 1]	[−1 −1 1]	[−1 0 0]	[1 0 0]	[2 1 3]	[0 1 3]
[uvw]Bi	[2 0 −2 −1]	[1 −1 0 −1]	[0 −2 2 −1]	[2 0 −2 −1]	[2 −1 −1 0]	[2 −2 0 1]	[−2 2 0 −1]	[−1 2 −1 0]	[0 2 −2 1]
d[uvw]Sn (Å)	3.324	6.047	2.801	3.324	4.422	2.916	2.917	4.044	2.801
d[uvw]Bi (Å)	3.286	4.746	3.286	3.286	4.546	3.286	3.286	4.546	3.286
*θ* (°)	0	0.175	0.332	0	4.965	2.464	0	2.393	2.464
*δ* (%)	14.4			5.2			12.4		

Cu_6_Sn_5_/(Bi) OR	(0 1 −1 1)Cu_6_Sn_5_//(0 1 −1 2)Bi			(0 0 0 1)Cu_6_Sn_5_//(0 0 0 1)Bi					
[uvw]Cu6Sn5	[1 −1 0 1]	[2 −1 −1 0]	[1 0 −1 −1]	[2 −1 −1 0]	[1 0 −1 0]	[1 1 −2 0]			
[uvw]Bi	[2 −2 0 1]	[2 −1 −1 0]	[2 0 −2 −1]	[2 −1 −1 0]	[1 0 −1 0]	[1 1 −2 0]			
d[uvw]Cu6Sn5 (Å)	2.946	4.192	2.946	4.192	7.261	4.192			
d[uvw]Bi (Å)	3.286	4.546	3.286	4.546	7.874	4.192			
*θ* (°)	0	1.594	3.189	0	0	0			
*δ* (%)	9.6			7.8					

**Figure 8:**
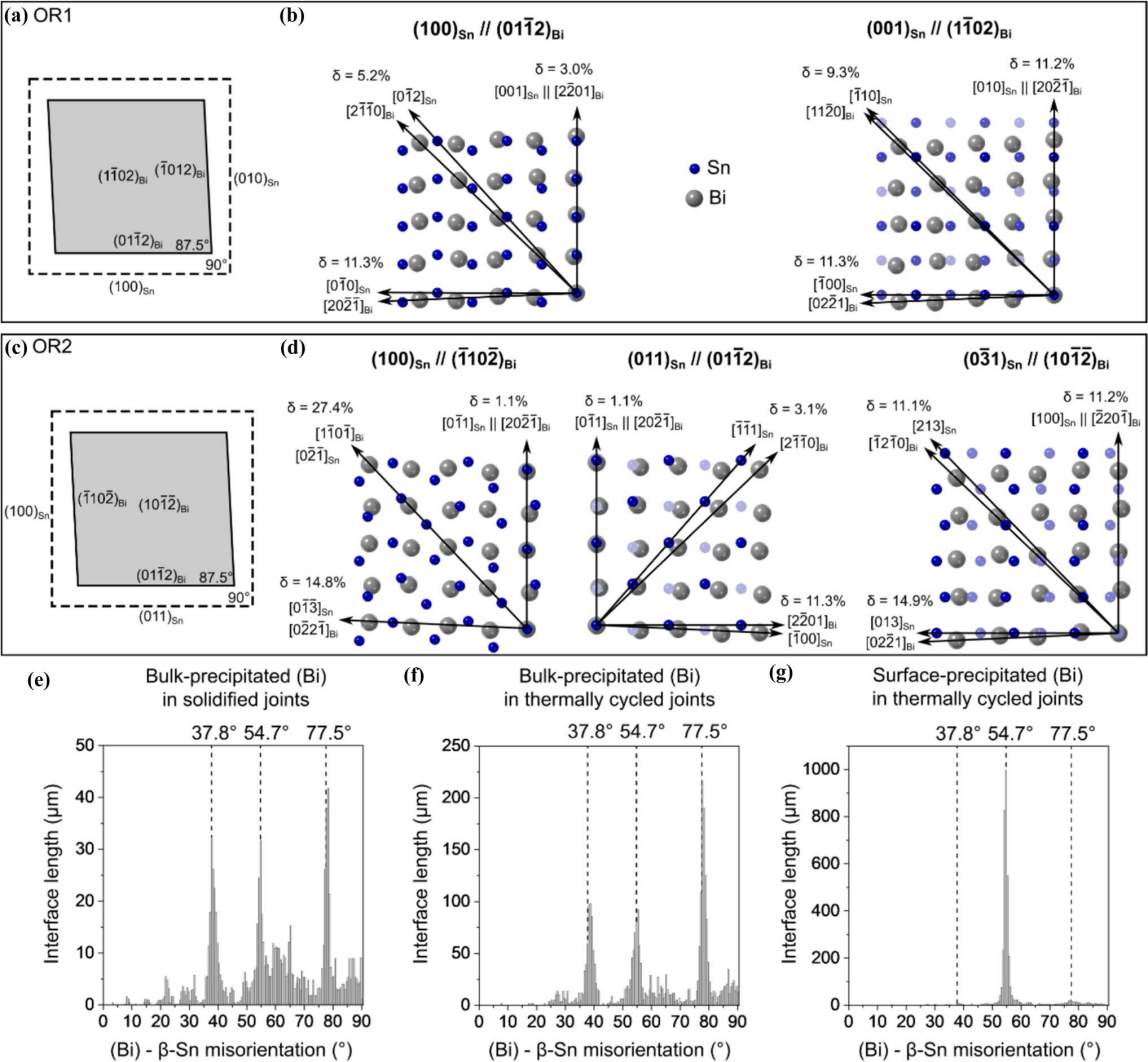
(Bi)/*β*-Sn interfaces in OR1 and OR2. **a**, **c** Schematics showing the angular differences between planes in OR1 and OR2. Only one member of ${(01\bar{1 }2)}_{\left(\mathrm{Bi}\right)}$ can be parallel with a low index plane of *β*-Sn for unstrained crystals in each OR. **b** Atomic match for the ${(100)}_{\beta \mathrm{Sn}}{\parallel (01\bar{1 }2)}_{\left(\mathrm{Bi}\right)}$ and ${(001)}_{\beta \mathrm{Sn}}{\parallel (1\bar{1 }02)}_{\left(\mathrm{Bi}\right)}$ interfaces in OR1. **d** Atomic match for the ${(100)}_{\beta \mathrm{Sn}}{\parallel (\bar{1 }10\bar{2 })}_{\left(\mathrm{Bi}\right)}$, ${(011)}_{\beta \mathrm{Sn}}{\parallel (01\bar{1 }2)}_{\left(\mathrm{Bi}\right)}$, and ${(0\bar{3 }1)}_{\beta \mathrm{Sn}}{\parallel (10\bar{1 }\bar{2 })}_{\left(\mathrm{Bi}\right)}$ interfaces in OR2a. **e**–**g** (Bi)/*β*-Sn misorientation (MO) histograms for (Bi)/*β*-Sn interfaces of (e) bulk-precipitated (Bi) in soldered-then-stored joints, **f** bulk-precipitated (Bi) in thermally-cycled-then-stored joints, and **g** surface-precipitated (Bi) in thermally-cycled-then-stored joints. In **e**–**g**, note that the theoretical misorientations are OR1 = 54.7°, OR2a = 77.5°, and OR2b = 37.8°.

For OR1, Table 3 shows that the ${(100)}_{\beta \mathrm{Sn}}{\parallel (01\bar{1 }2)}_{\left(Bi\right)}$ and ${(010)}_{\beta \mathrm{Sn}}\parallel {(\bar{1 }012)}_{\left(\mathrm{Bi}\right)}$ interfaces have a lower disregistry (*δ* ~ 6.5%) than the ${(001)}_{\beta \mathrm{Sn}}{\parallel (1\bar{1 }02)}_{\left(\mathrm{Bi}\right)}$ interface (*δ* ~ 10.6%). The atomic model in Fig. 8b shows the Sn and Bi atoms in an unstrained ${(100)}_{\beta \mathrm{Sn}}{\parallel (01\bar{1 }2)}_{\left(\mathrm{Bi}\right)}$ interface, and highlights that the lowest disregistry (*δ* ~ 3.2%) is in the ${[001]}_{\beta \mathrm{Sn}}{\parallel [2\bar{2 }01]}_{\left(\mathrm{Bi}\right)}$ direction. This is consistent with (Bi) particles with OR1 exhibiting ${\{100\}}_{\beta \mathrm{Sn}}{\parallel \{01\bar{1}2\} }_{\left(Bi\right)}$ as the largest habit planes (Fig. 4f) and being elongated along the ${\langle 001\rangle }_{\beta Sn}{\parallel \langle 2\bar{2}01\rangle }_{\left(\mathrm{Bi}\right)}$ axis [51]. Note that some Sn atoms in the ${(001)}_{\beta Sn}{\parallel (1\bar{1 }02)}_{\left(Bi\right)}$ interface are out-of-plane, and are plotted as faded atoms in Fig. 8b, which further reduces the coherency of the minor ${(001)}_{\beta \mathrm{Sn}}{\parallel (1\bar{1 }02)}_{\left(\mathrm{Bi}\right)}$ interface.

The 87.5° angle between adjacent ${\left\{01\bar{1}2\right\} }_{\left(\mathrm{Bi}\right)}$ planes should also be considered. Figure 8a presents a schematic with the dashed square representing ${\left\{100\right\}}_{\beta \mathrm{Sn}}$ planes and a grey rhombus representing ${\left\{01\bar{1}2\right\} }_{\left(\mathrm{Bi}\right)}$ planes. The ${(100)}_{\beta \mathrm{Sn}}$ is parallel with the ${(01\bar{1 }2)}_{\left(\mathrm{Bi}\right)}$, but the ${(010)}_{\beta \mathrm{Sn}}$ is misaligned from the ${(\bar{1 }012)}_{\left(\mathrm{Bi}\right)}$ due to the 87.5° angle of (Bi). With the 87.5° misalignment, the non-overlapped ${\left\{100\right\}}_{\beta \mathrm{Sn}}$/${\left\{01\bar{1}2\right\} }_{\left(\mathrm{Bi}\right)}$ interface is less coherent and should contain a significantly higher density of dislocations in order to accommodate the larger lattice mismatch between the two phases. Thus, the non-overlapped ${\left\{100\right\}}_{\beta \mathrm{Sn}}$/${\left\{01\bar{1}2\right\} }_{\left(\mathrm{Bi}\right)}$ interface is expected to have higher interfacial energy than the overlapped ${\{100\}}_{\beta \mathrm{Sn}}{\parallel \{01\bar{1}2\} }_{\left(\mathrm{Bi}\right)}$ interface. This explains why, in Fig. 4, the non-overlapped ${\left\{100\right\}}_{\beta \mathrm{Sn}}$/${\left\{01\bar{1}2\right\} }_{\left(\mathrm{Bi}\right)}$ interface tends to be shorter and more curved than the overlapped ${\{100\}}_{\beta \mathrm{Sn}}{\parallel \{01\bar{1}2\} }_{\left(\mathrm{Bi}\right)}$ interface.

For OR2, Table 3 shows the planar disregistries of OR2a. Note that the values are similar between OR2a and OR2b so only OR2a is shown. The ${(011)}_{\beta \mathrm{Sn}}\parallel {(01\bar{1 }2)}_{\left(\mathrm{Bi}\right)}$ interface exhibits a significantly lower disregistry *δ* ~ 5.2%) than the ${(100)}_{\beta \mathrm{Sn}}\parallel {(\bar{1 }10\bar{2 })}_{\left(\mathrm{Bi}\right)}$ and ${(0\bar{3 }1)}_{\beta \mathrm{Sn}}\parallel {\left(10\bar{1 }\bar{2 }\right)}_{\left(\mathrm{Bi}\right)}$ interfaces in OR2. This is illustrated for the ${(011)}_{\beta \mathrm{Sn}}\parallel {(01\bar{1 }2)}_{\left(\mathrm{Bi}\right)}$ by the atomic model inFig. 8d, where Sn and Bi atoms are relatively well-matched in a near-square array, and particularly along the ${[0\bar{1 }1]}_{\beta \mathrm{Sn}}{\parallel [20\bar{2 }\bar{1 }]}_{\left(\mathrm{Bi}\right)}$ direction. This is consistent with the result that (Bi) particles with OR2 exhibited ${\{011\}}_{\beta \mathrm{Sn}}{\parallel \{01\bar{1}2\} }_{\left(\mathrm{Bi}\right)}$ as a long and straight interface (Fig. 7d) and were elongated along an ${\langle 011\rangle }_{\beta \mathrm{Sn}}{\parallel \langle 2\bar{2}01\rangle }_{\left(\mathrm{Bi}\right)}$ axis (Fig. 7b). The 90° versus 87.5° angular mismatch shown schematically in Fig. 8c is likely to further reduce the coherency on the ${\{100\}}_{\beta \mathrm{Sn}}$ and ${\{0\bar{3}1\} }_{\beta \mathrm{Sn}}$ interfaces.

Comparing the disregistries of OR1 and OR2 (Table 3), we see that OR1 has two low-disregistry interfaces, ${(100)}_{\beta \mathrm{Sn}}{\parallel (01\bar{1 }2)}_{\left(\mathrm{Bi}\right)}$ and ${(010)}_{\beta \mathrm{Sn}}\parallel {(\bar{1 }012)}_{\left(\mathrm{Bi}\right)}$, while OR2 only has one low-disregistry interface, ${(011)}_{\beta Sn}\parallel {(01\bar{1 }2)}_{\left(\mathrm{Bi}\right)}$. This suggests that (Bi) particles with OR1 may have a lower total interfacial energy than those with OR2. Additionally, although the ${(011)}_{\beta Sn}\parallel {(01\bar{1 }2)}_{\left(\mathrm{Bi}\right)}$ interface of OR2 has a relatively low planar disregistry (Table 3), some Sn atoms are slightly out-of-plane due to the zig-zag nature of the ${(011)}_{\beta \mathrm{Sn}}$ plane (plotted as faded atoms in Fig. 8d), which may contribute to reduced coherency of the ${\{011\}}_{\beta \mathrm{Sn}}\parallel {\{01\bar{1}2\} }_{\left(\mathrm{Bi}\right)}$ interface in OR2.

The preference in ORs between (Bi) and *β*-Sn was found to be significantly different for (Bi) particles that precipitated in the bulk solder versus those that precipitated on the free surface as stated in Table 2 and overviewed in Fig. 8e–g. (Bi)–*β*-Sn misorientation (MO) histograms are used to present statistical data, and the cross sections of at least four joints are combined in each histogram in Fig. 8e–g. The ORs can be deduced from the MO angle, which is 54.7° for OR1, 37.8° for OR2b and 77.5° for OR2a.

Figure 8g shows the (Bi)-*β*-Sn MO histogram for surface-precipitated (Bi) in thermally cycled joints, where only one peak at MO = 54.75°, corresponding to OR1, is visible, and the peaks corresponding to OR2 (MO = 37.8° and 77.5°) are absent. The data were obtained from surface-precipitated (Bi) acquired one month after polishing. This indicates that, during one month of precipitation and coarsening of (Bi) on the surface, (Bi) particles with OR1 outcompeted those with OR2. This result is consistent with Fig. 2 and Ref. [51, 53], and can be attributed to the higher overall disregistry of OR2 compared to OR1 (Table 3).

However, a contrary result was observed for (i) bulk-precipitated (Bi) in joints that had been soldered-then-stored (Fig. 8e) and (ii) bulk-precipitated (Bi) in thermally-cycled-then-stored joints (Fig. 8f). In both cases, (Bi) with OR1, OR2a and OR2b are all present and the peaks of OR2a and OR2b are higher than that of OR1, indicating that these bulk-precipitated (Bi) particles exhibited a preference for OR2. Calculating the number of (Bi) particles with each OR reveals that, for bulk-precipitated (Bi), the number of particles with OR2 is at least twice that of those with OR1. Two factors that are expected to affect precipitation in the bulk that are less important for surface precipitation are (i) the strain energy associated with (Bi) precipitation and (ii) the anisotropic diffusivity of Bi in *β*-Sn, which affect the thermodynamic driving force and kinetics of precipitation, respectively.

Precipitation in the bulk requires a significant strain energy associated with the ~ 30% volumetric expansion misfit strain induced when (Bi) precipitates within the bulk of *β*-Sn (see SI-Fig. 4 in the Supplementary Information), and both volumetric misfit strain energy and interfacial energy will affect the driving force for precipitation. It is well known that the shape of precipitates affects their misfit strain to the matrix [74, 75], and further work is required to quantify the detailed 3D shapes of (Bi) particles and link these quantitatively with strain energy to test if particles with OR2 have a lower volumetric misfit strain energy and, therefore, an advantage over OR1 during precipitation in the bulk. At this stage, a general comment can be made based on elastic constants of *β*-Sn along the elongated directions of the different plates. The elastic modulus of *β*-Sn along ${\langle 001\rangle }_{\beta \mathrm{Sn}}$ is more than twice that along ${\langle 100\rangle }_{\beta \mathrm{Sn}}$ [76]. Therefore, the elongation of (Bi) particles with OR1 along ${\langle 001\rangle }_{\beta \mathrm{Sn}}$ generates a larger elastic strain energy than the elongation of (Bi) particles with OR2 along ${\langle 011\rangle }_{\beta \mathrm{Sn}}$. This smaller strain energy for precipitates elongating along ${\langle 011\rangle }_{\beta \mathrm{Sn}}$ gives OR2 an advantage over OR1 for precipitation in the bulk.

The anisotropic diffusivity of Bi in *β*-Sn can also play a role, as Delhaise et al. [77, 78] reported that the diffusivity of Bi in *β*-Sn increases with increasing angle between ${\langle 001\rangle }_{\beta \mathrm{Sn}}$ and the direction of diffusion. (Bi) particles with OR1 are elongated along ${\langle 001\rangle }_{\mathrm{Sn}}$ [51], while (Bi) particles with OR2 are elongated along ${\langle 011\rangle }_{\beta \mathrm{Sn}}$ (Fig. 7b). Thus, the growth of bulk (Bi) particles with OR1 and OR2 is predominantly by the diffusion of Bi along ${\langle 001\rangle }_{\beta \mathrm{Sn}}$ and ${\langle 011\rangle }_{\beta \mathrm{Sn}}$, respectively. According to the diffusivity data by Delhaise et al. [77, 78], the diffusivity of Bi along ${\langle 001\rangle }_{\beta \mathrm{Sn}}$ is ~ 3 × 10^–15^ cm^2^s^−1^, while that along ${\langle 011\rangle }_{\beta \mathrm{Sn}}$ is ~ 4.1 × 10^–14^ cm^2^s^−1^. With over ten times faster diffusion of Bi along ${\langle 011\rangle }_{\beta \mathrm{Sn}}$ than along ${\langle 001\rangle }_{\beta \mathrm{Sn}}$, (Bi) particles with OR2 have a kinetic advantage over those with OR1, enabling OR2 to compete with OR1 during precipitation and coarsening in the bulk, Fig. 8e, f. The anisotropy also affected the kinetics of surface precipitation although in a different manner because, in surface precipitation, the relevant factor is Bi diffusion towards the free surface as presented in the next section.

#### *Crystallographic aspects of the precipitation of (*Bi*)*

**Figure 9:**
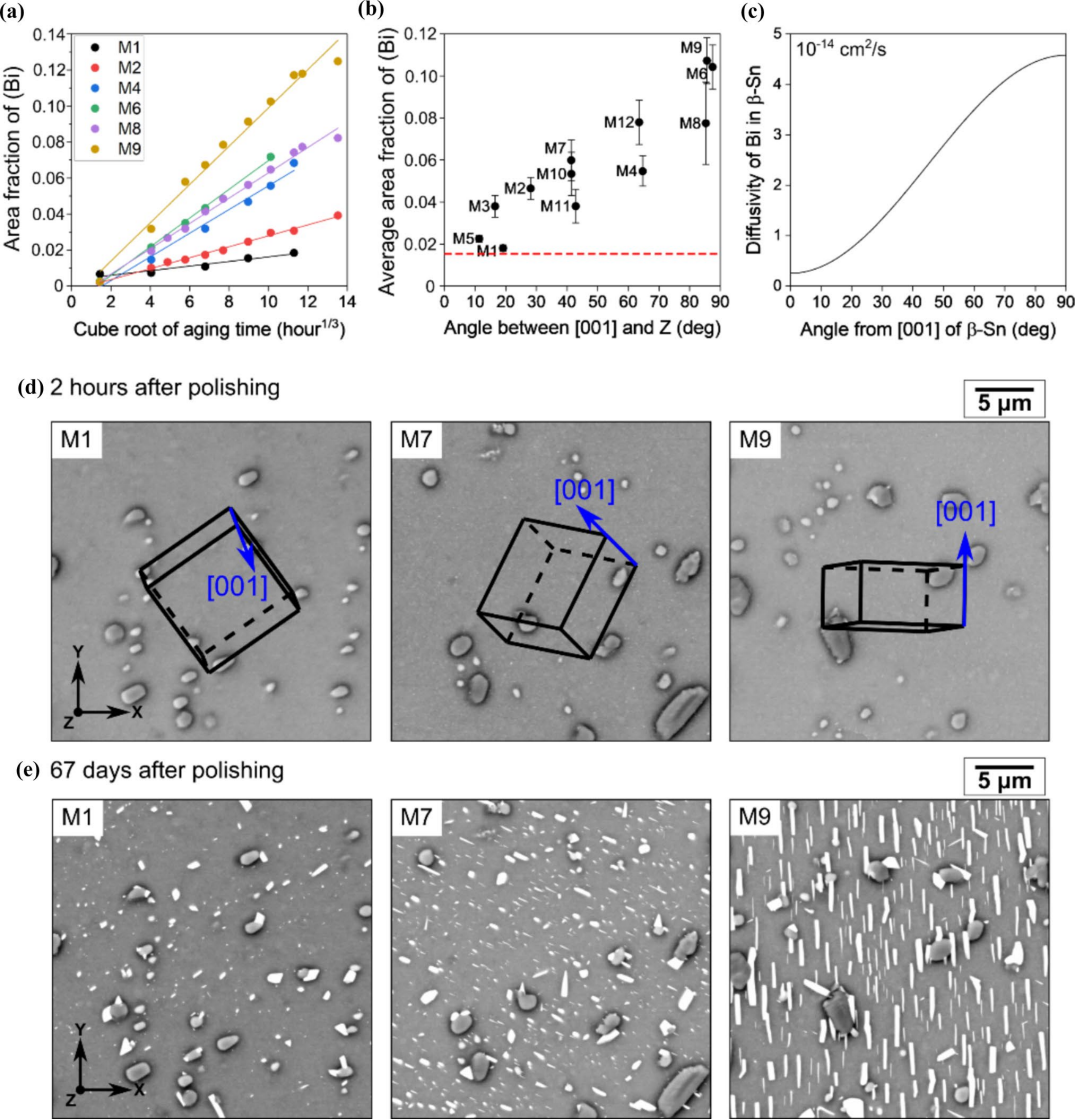
Effect of *β*-Sn orientation on the surface precipitation of (Bi). **a** The area fractions of (Bi) in six joints versus cube root of ageing time after polishing. **b** The average (Bi) area fractions of 12 joints 67 days after polishing, versus the angle between ${\langle 001\rangle }_{\beta \mathrm{Sn}}$ and surface normal *Z*. The red dashed line marks the equilibrium volume fraction of (Bi) at 23 °C [80]. **c** Diffusivity of Bi in *β*-Sn at 23 °C versus the angle from ${\langle 001\rangle }_{\beta \mathrm{Sn}}$, data from Ref. [78]. The BSE images show the *β*-Sn orientations and microstructures of three joints **d** 2h and **e** 67 days after polishing. This example is from surface-precipitated (Bi) in a thermally-cycled-then-stored 84CTBGA joint.

The *β*-Sn grain orientation was found to affect the kinetics of (Bi) surface precipitation as overviewed in Fig. 9. The area fractions of (Bi) were measured on the surface of 12 thermally cycled Sn-2.25Ag-0.5Cu-6Bi joints (M1–M12) in the outer row of an 84CTBGA package. The measurements were conducted on single-grained joints such as Fig. 2b, minimising the influence of *β*-Sn grain boundaries. Figure 9a shows the area fractions of (Bi) in six joints measured by time-lapse imaging on a single area containing > 500 (Bi) particles during room-temperature ageing for up to 67 days using the method presented in ref. [53]. For all six joints, the area fractions of (Bi) increased linearly with the cube root of ageing time, consistent with previous studies [41, 51]. However, the precipitation rate, i.e. the slope of the fitted lines in Fig. 9a, varies from joint to joint. For example, the precipitation rate constant of joint M9 is ~ 0.0106 h^−1/3^, about six times that of joint M1 (~ 0.0013 h^−1/3^).

Figure 9b shows the mean area fraction of (Bi) for each joint at 67 days after polishing, versus the angle between the ${\langle 001\rangle }_{\beta \mathrm{Sn}}$ of the matrix and the surface normal *Z*. Assuming the area fraction of (Bi) was ~ 0% for all joints in the as-polished states, the distribution in Fig. 9b indicates that (Bi) precipitated on the surface more slowly for joints with a smaller angle between ${\langle 001\rangle }_{\beta \mathrm{Sn}}$ and the surface normal. This trend is clearly illustrated in the examples shown in Fig. 9d, e, where the SEM images were taken from the same areas two hours and 67 days after polishing. After 67 days (Fig. 9e), joint M9, with ${\langle 001\rangle }_{\beta \mathrm{Sn}}$ lying nearly on the surface plane, showed the most significant (Bi) precipitation. In contrast, joint M1, with ${\langle 001\rangle }_{\beta \mathrm{Sn}}$ nearly perpendicular to the surface plane, exhibited much less (Bi) precipitation. The BSE images of all 12 joints can be found in SI-Fig. 5 in Supplementary Information.

The dependence of (Bi) surface precipitation on *β*-Sn orientation is partly associated with the anisotropic diffusivity of Bi in *β*-Sn. As shown in Fig. 9c, the calculated diffusivity of Bi in *β*-Sn increases as the angle from ${\langle 001\rangle }_{\beta \mathrm{Sn}}$ increases [78], indicating that Bi can diffuse faster from the bulk towards the free surface when the surface normal has a larger angle with the ${\langle 001\rangle }_{\beta \mathrm{Sn}}$ of the matrix. Note that, the diffusivity values in Fig. 9c were calculated based on Equations [78, 79]:

$$D_{\theta } = D_{\parallel } \cos^{2} \theta + D_{ \bot } \sin^{2} \theta$$

where ${D}_{\theta }$ is the diffusivity of Bi in *β*-Sn as a function of the angle $\theta$ from the *c*-axis. ${D}_{\parallel }$ and ${D}_{\perp }$ are the diffusivities parallel and perpendicular to the *c*-axis as reported in Ref. [78]. However, it should be noted that the precipitation process occurs at a freshly polished free surface. In this context, additional mass transport pathways, e.g. surface diffusion or pipe diffusion along near-surface defects, may also play a role. These mechanisms may exhibit kinetic behaviours and anisotropies that differ substantially from bulk diffusion, potentially influencing the observed precipitation kinetics. As such, the present interpretation, which primarily considers bulk diffusion, may represent a simplified description of a more complex scenario. A more detailed investigation of near-surface transport processes would therefore be valuable in clarifying their contribution to the overall kinetics.

#### Orientation relationships between (Bi) and IMCs

**Figure 10:**
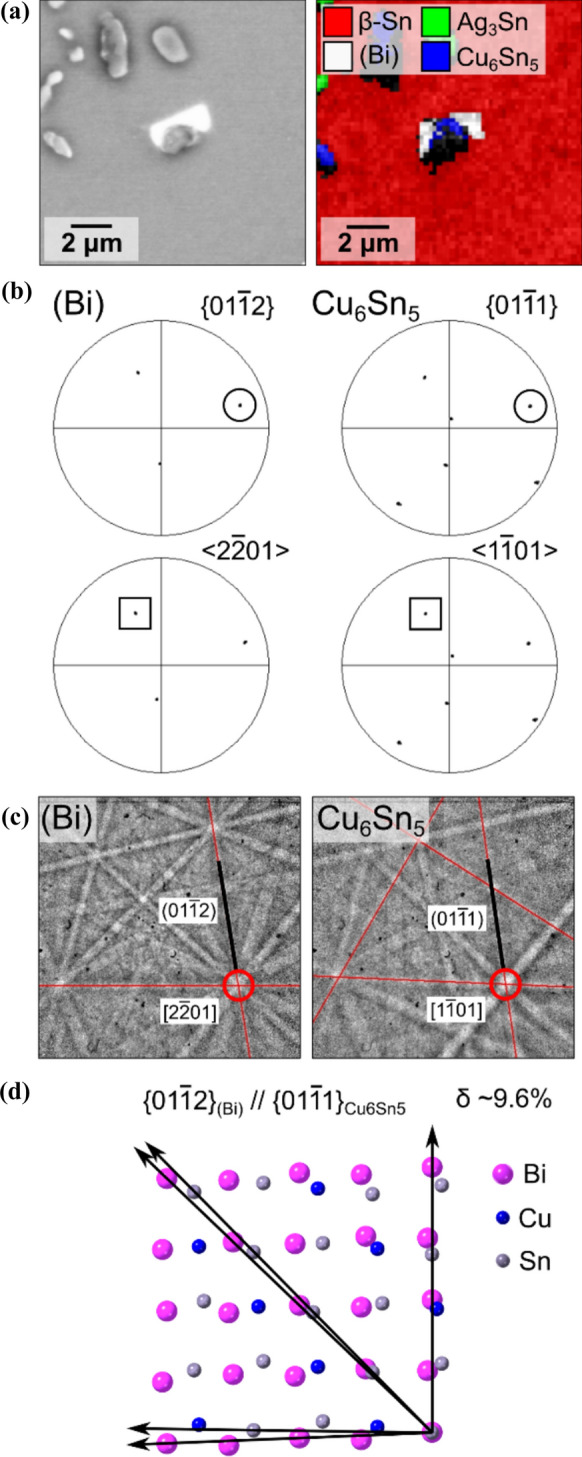
A (Bi) and Cu_6_Sn_5_ particle with an orientation relationship. **a** BSE image and EBSD phase map. **b** EBSD pole figures. **c** Kikuchi patterns with ${\{01\bar{1}2\} }_{(\mathrm{Bi})}$ and ${\{01\bar{1}1\} }_{\mathrm{Cu}6\mathrm{Sn}5}$ bands marked by red lines and ${\langle 2\bar{2}01\rangle }_{(\mathrm{Bi})}$ and ${\langle 1\bar{1}01\rangle }_{\mathrm{Cu}6\mathrm{Sn}5}$ poles with red circles. **d** Atomic match for the ${\left\{01\bar{1}1\right\} }_{\mathrm{Cu}6\mathrm{Sn}5}{\langle 1\bar{1}01\rangle }_{\mathrm{Cu}6\mathrm{Sn}5}\parallel {\left\{01\bar{1}2\right\} }_{\left(\mathrm{Bi}\right)}{\langle 2\bar{2}01\rangle }_{(\mathrm{Bi})}$ interface. This example is from a soldered-then-stored 192CABGA joint.

In many cases, (Bi) precipitates shared an interface with Cu_6_Sn_5_ particles within the solder. In the cases in Figs. 6a, 7b such (Bi) precipitates had OR1 or OR2a/b with the *β*-Sn and no OR with the Cu_6_Sn_5_. In other cases, the (Bi) shared an OR with Cu_6_Sn_5_ and not with the surrounding *β*-Sn. Figure 10a shows an example in a soldered-then-stored 192CABGA joint. As shown in Fig. 10b, EBSD analysis gave the following orientation relationship between the two phases (using the hexagonal indices of the *η*-Cu_6_Sn_5_ phase):

$$\left\{ {01\bar{1}1} \right\}_{{{\mathrm{Cu6Sn5}}}} \parallel \left\{ {01\bar{1}2} \right\}_{{\left( {{\mathrm{Bi}}} \right)}} \;{\mathrm{and}}\;\left\langle {1\bar{1}01} \right\rangle _{{{\mathrm{Cu6Sn5}}}} \parallel \left\langle {2\bar{2}01} \right\rangle _{{\left( {{\mathrm{Bi}}} \right)}}$$

**Figure 11:**
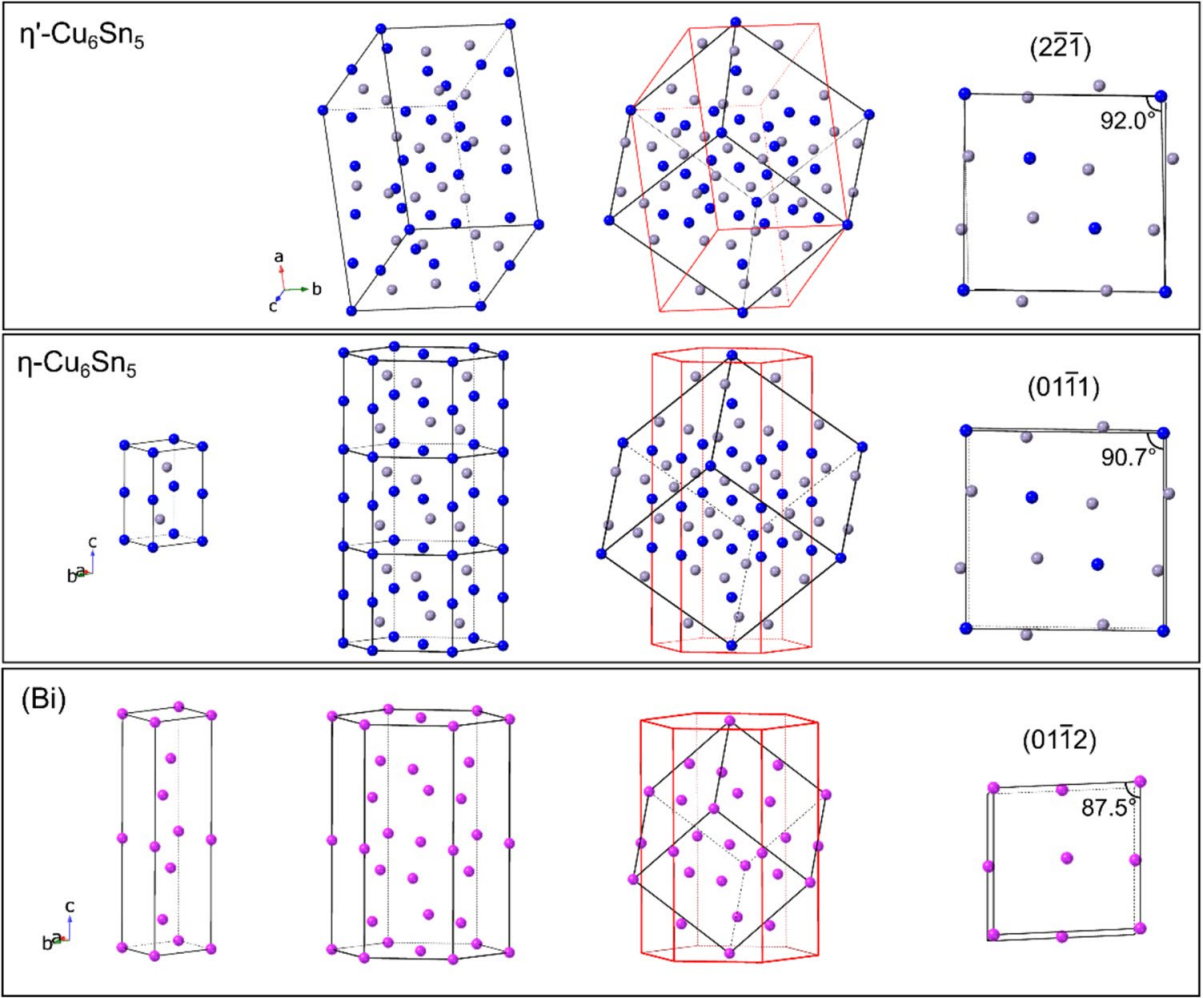
Transformations of *η*′-Cu_6_Sn_5_, *η*-Cu_6_Sn_5_, and (Bi) from their standard monoclinic/hexagonal unit cells to a pseudo-cubic unit cell. The first three columns have the same viewing direction. The fourth column is viewed perpendicular to a $(100)$ face of the pseudo-cubic unit cells. The structures and lattice parameters in Table 1 were used.

The Kikuchi patterns in Fig. 10c further confirm the overlap between the ${\left\{01\bar{1}1\right\} }_{\mathrm{Cu}6\mathrm{Sn}5}$ and ${\left\{01\bar{1}2\right\} }_{\left(\mathrm{Bi}\right)}$ bands and ${\langle 1\bar{1}01\rangle }_{\mathrm{Cu}6\mathrm{Sn}5}$ and ${\langle 2\bar{2}01\rangle }_{(\mathrm{Bi})}$ poles. Note that the structure of (Bi) can be described by a pseudo-cubic unit cell bounded by ${\left\{01\bar{1}2\right\} }_{\left(\mathrm{Bi}\right)}$ planes (the FCR unit cell in Table 1), and the structure of Cu_6_Sn_5_ is also pseudo-cubic [81–83] and can be described by a unit cell bounded by ${\left\{01\bar{1}1\right\} }_{\mathrm{Cu}6\mathrm{Sn}5}$ planes, as shown in Fig. 11. Thus, this Cu_6_Sn_5_-(Bi) OR can be approximated as a pseudo-cube-on-cube relationship. However, this neglects some important details of the hexagonal/rhombohedral symmetry.

Examining numerous (Bi) particles, two ORs were measured between Cu_6_Sn_5_ and (Bi) which can be written as follows using the hexagonal indices of the *η*-Cu_6_Sn_5_ phase.

ORa $$\left\{ {01\bar{1}1} \right\}_{{{\mathrm{Cu6Sn5}}}} \parallel \left\{ {01\bar{1}2} \right\}_{{\left( {{\mathrm{Bi}}} \right)}} \;{\mathrm{and}}\;\left\langle {1\bar{1}01} \right\rangle _{{{\mathrm{Cu6Sn5}}}} \parallel \left\langle {2\bar{2}01} \right\rangle _{{\left( {{\mathrm{Bi}}} \right)}}$$

ORb $$\left\{ {01\bar{1}1} \right\}_{{{\mathrm{Cu6Sn5}}}} \parallel \left\{ {\bar{1}012} \right\}_{{\left( {{\mathrm{Bi}}} \right)}} \;{\mathrm{and}}\;\left\langle {1\bar{1}01} \right\rangle _{{{\mathrm{Cu6Sn5}}}} \parallel \left\langle {2\bar{2}01} \right\rangle _{{\left( {{\mathrm{Bi}}} \right)}}$$

Both ORs involve a member of ${\left\{01\bar{1}2\right\} }_{\left(\mathrm{Bi}\right)}$ parallel with ${\left\{01\bar{1}1\right\} }_{\mathrm{Cu}6\mathrm{Sn}5}$ and a member of ${\langle 2\bar{2}01\rangle }_{(\mathrm{Bi})}$ parallel with ${\langle 1\bar{1}01\rangle }_{\mathrm{Cu}6\mathrm{Sn}5}$ but they are different ORs because $\{0001\}$ of (Bi) is parallel with the $\{0001\}$ of Cu_6_Sn_5_ in ORa (Fig. 13b) but not in ORb (Fig. 13c). Considering the ~ 87.5° angle between ${\left\{01\bar{1}2\right\} }_{\left(\mathrm{Bi}\right)}$ planes and the ~ 90.5° angle between ${\left\{01\bar{1}1\right\} }_{\mathrm{Cu}6\mathrm{Sn}5}$ planes, both ORa and ORb contain 12 (Bi) orientation variants each, as displayed in Fig. 13a–c in the Appendices. From 29 pairs of measured (Bi)/Cu_6_Sn_5_ particles, it was found that ~ 62% had ORa, i.e. with ${\left\{0001\right\}}_{\left(\mathrm{Bi}\right)}\parallel {\left\{0001\right\}}_{\mathrm{Cu}6\mathrm{Sn}5}$, indicating a mild preference for ORa over ORb.

During slow cooling after soldering and during thermal cycling from -55/125 °C, the Cu6Sn5 phase is likely to transform into the low temperature equilibrium *η*′-Cu6Sn5 phase [84]. Figures 11, 14 further present the (Bi)/Cu6Sn5 OR using the monoclinic *η*′-Cu6Sn5 structure. Comparing Fig. 13b, c with Fig. 14b–m we see that the orientations of all 24 (Bi) variants remain essentially unchanged between hexagonal *η*-Cu6Sn5 and monoclinic *η*′-Cu6Sn5. However, there is a change from two ORs with 12 (Bi) variants each for hexagonal *η*-Cu6Sn5, to 12 ORs with two (Bi) variants each for monoclinic *η*′-Cu6Sn5. This is caused by the reduction in symmetry from hexagonal to monoclinic. We did not explore whether there is any preference among the 12 ORs with monoclinic *η*′-Cu6Sn5. Due to the very small metric distortion between hexagonal *η* and monoclinic *η*′, the atomic matching across (Bi)-Cu6Sn5 interfaces is only subtly different for *η*′ compared with *η*.

**Figure 12:**
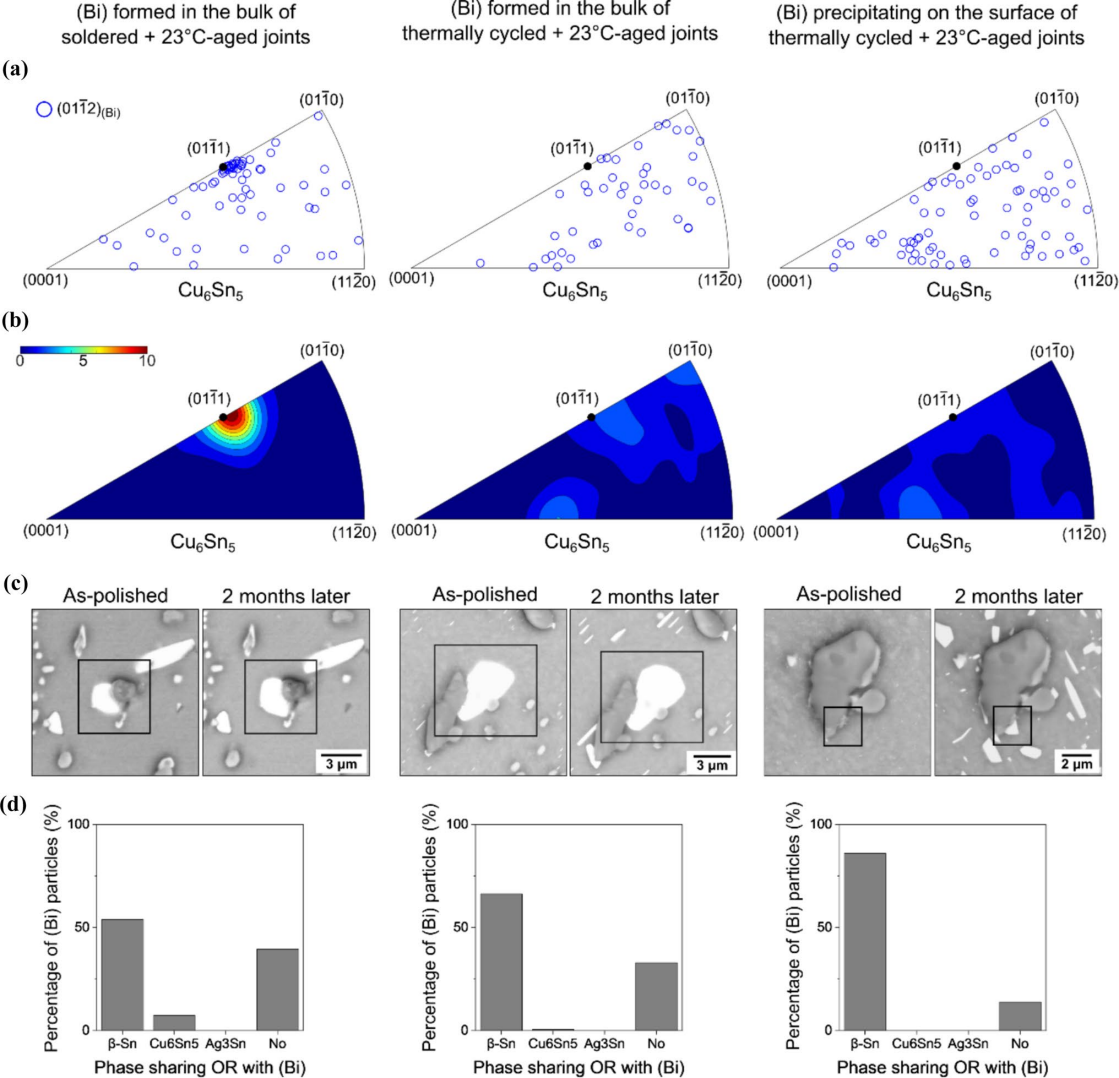
Reproducibility of Cu_6_Sn_5_/(Bi) ORs for three groups of (Bi) particles: (Bi) in the bulk of soldered-then-stored joints (left); (Bi) in the bulk of thermally-cycled-then-stored joints (middle); and (Bi) precipitating on the free surface of thermally-cycled-then-stored joints after polishing (right). **a** IPF, **b** contour IPF, **c** BSE images, and **d** column charts summarising the fractions of (Bi) particles with an OR to *β*-Sn, Cu_6_Sn_5_, Ag_3_Sn, or without OR.

Figure 12 demonstrates that the Cu₆Sn₅/(Bi) orientation relationship occurred only for bulk-precipitated (Bi) particles in soldered-then-stored solder joints, and not for (Bi) particles in thermally cycled joints.

**Figure 13:**
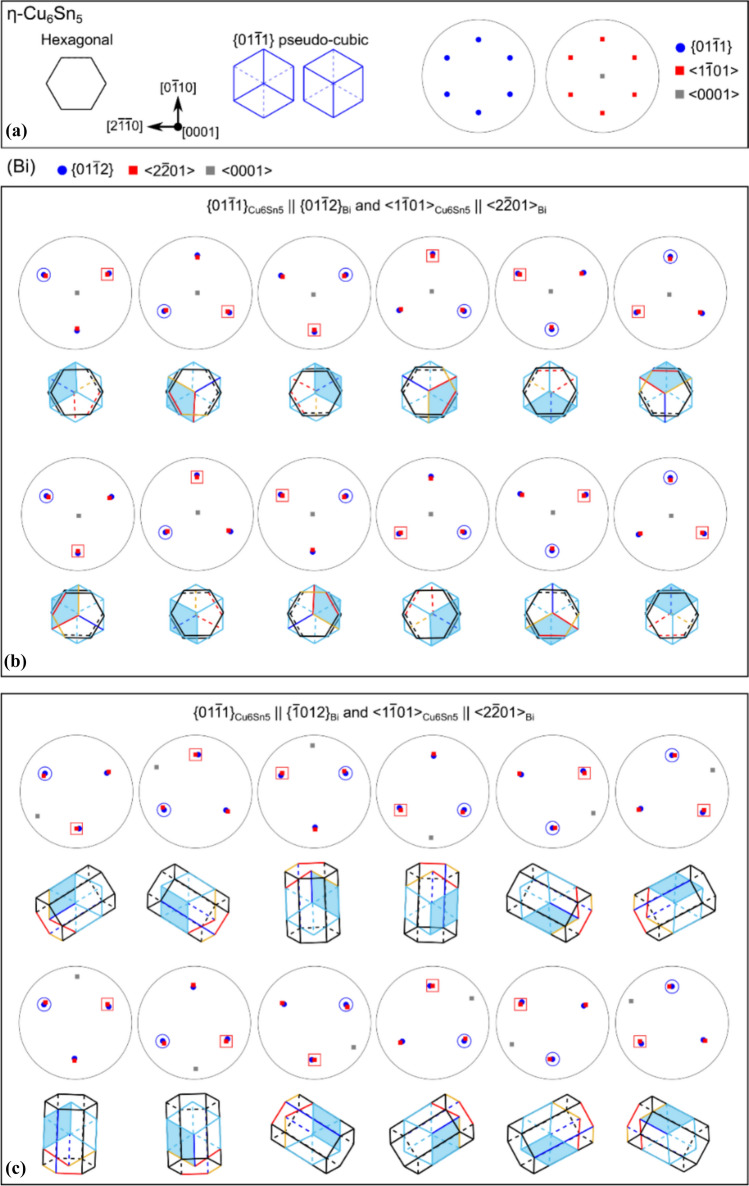
Calculated orientation variants of (Bi) in the ORs with hexagonal *η*-Cu_6_Sn_5_. **a** a single orientation of $\eta$-Cu_6_Sn_5_. **b** 12 (Bi) variants of ORa. **c** 12 (Bi) variants of ORb. **a** is plotted with the $\eta$-Cu_6_Sn_5_ c-axis pointing out of the page and $[0\bar{1 }10]$ pointing up. The blue wireframes are the orientations of two pseudo-cubic unit cells, each bounded by six of the 12 $\left\{ {01\bar{1}1} \right\}$ planes and with internal angle *α* = 90.7° for the $\eta$-Cu_6_Sn_5_ lattice parameters in Table 1. In **b** and **c**, the wireframes of the hexagonal (Bi) unit cell are plotted in black and the pseudo-cubic face-centred rhombohedral (Bi) unit cells are plotted in cyan. The blue shaded plane is the $\left\{ {01\bar{1}2} \right\}$ of (Bi) that is parallel with a $\left\{ {01\bar{1}1} \right\}$ of $\eta$-Cu_6_Sn_5_. In the pole figures in **b** and **c**, the blue circles highlight which $\left\{ {01\bar{1}2} \right\}$ of (Bi) is parallel with a $\left\{ {01\bar{1}1} \right\}$ of $\eta$-Cu_6_Sn_5_; and the red squares highlight which $\langle 2\bar{2 }01\rangle$ of (Bi) is parallel with a $\langle 1\bar{1 }01\rangle$ of $\eta$-Cu_6_Sn_5._

**Figure 14:**
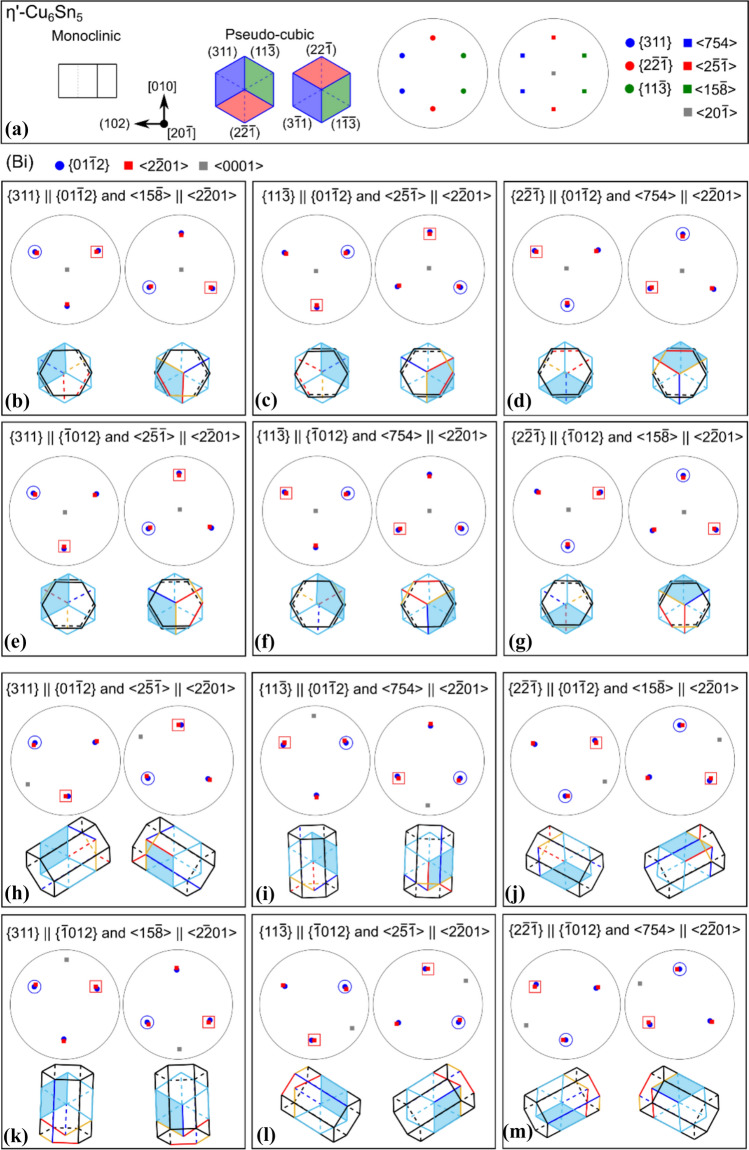
Calculated orientation variants of (Bi) in the ORs with monoclinic$\eta^{\prime}$-Cu_6_Sn_5_. **a** a single orientation of $\eta^{\prime}$-Cu_6_Sn_5_. **b**–**m** twelve black boxes, each representing a different OR, containing 2 (Bi) orientation variants each. **a** is plotted with the $\eta^{\prime}$-Cu_6_Sn_5_ $[20\bar{1 }]$ pointing out of the page and $[010]$ pointing up. The coloured wireframes are the orientations of two pseudo-cubic unit cells, each bounded by two$\left\{ {311} \right\}$ , two $\left\{ {22\bar{1}} \right\}$ and two $\left\{ {11\bar{3}} \right\}$ and with internal angle *α* = 92° for the $\eta^{\prime}$-Cu_6_Sn_5_ lattice parameters in Table 1. In **b**–**m** each letter and box contains one OR with two (Bi) variants. The 24 (Bi) orientations in **b–m** are sorted and presented in the same way as those for hexagonal *η*-Cu_6_Sn_5_ in Figure 13b, c.

The columns in Fig. 12 split the (Bi) into three groups: (left) (Bi) formed in the bulk of soldered-then-stored joints; (middle) (Bi) formed in the bulk of thermally-cycled-then-stored joints; and (right) (Bi) precipitating on the free surface of thermally-cycled-then-stored joints after polishing. Fig. 12a, b summarises the orientations of (Bi) with respect to Cu_6_Sn_5_, with > 40 (Bi) particles contained in each inverse pole figure. To avoid the influence of *β*-Sn/(Bi) ORs, only the (Bi) particles without a simple OR with *β*-Sn were chosen for analysis. For (Bi) in the bulk of soldered-then-stored joints (left), the inverse pole figures display a preference for ${(01\bar{1 }2)}_{(\mathrm{Bi})}$ to be parallel with ${(01\bar{1 }1)}_{\mathrm{Cu}6\mathrm{Sn}5}$, consistent with ORa and ORb in Figs. 10, 13. In contrast, for (Bi) in the bulk of thermally-cycled-then-stored joints (middle) and surface-precipitated (Bi) (right), ${(01\bar{1 }2)}_{(\mathrm{Bi})}$ (blue circle) is near-uniformly distributed in the Cu_6_Sn_5_ inverse pole figures. This indicates no preference for ${(01\bar{1 }2)}_{(\mathrm{Bi})}$ to be parallel with any planes of Cu_6_Sn_5_, i.e. no OR between (Bi) and Cu_6_Sn_5_ for bulk-precipitated and surface-precipitated (Bi) particles in thermally-cycled-then-stored joints.

Thus, some (Bi) had an OR with Cu_6_Sn_5_ in soldered-then-stored joints but not in thermally-cycled-then-stored joints. A major difference between these two thermal histories is the phase transformation(s) that created the (Bi) phase. In soldered-then-stored joints, some (Bi) particles initially formed during solidification in a late eutectic reaction after eutectic Cu_6_Sn_5_ had formed (Fig. 1e), and some (Bi) precipitated in the solid state. In contrast, in thermally-cycled-then-stored joints, (Bi) dissolved and reprecipitated during temperature cycling (Fig. 1f) and, therefore, all (Bi) precipitated in the solid state for this thermal history. This observation implies that the (Bi) particles with an OR to Cu_6_Sn_5_ may have nucleated on Cu_6_Sn_5_ during eutectic solidification, whereas (Bi) particles without an OR to Cu_6_Sn_5_ precipitated in the solid state where they did not nucleate on Cu_6_Sn_5_. However, further work is required to test this tentative interpretation. For the (Bi) particles without an OR to Cu_6_Sn_5_, it is likely that solid-state-precipitated (Bi) came into contact with Cu_6_Sn_5_ particles during growth, instead of nucleating on them. Here, the driving force for (Bi) to grow into contact with Cu_6_Sn_5_ during coarsening is a reduction in total interfacial energy when replacing a (Bi)/*β*-Sn and a Cu_6_Sn_5_/*β*-Sn interface with a single (Bi)/Cu_6_Sn_5_ interface and thereby reducing interfacial area.

Finally, to consider the relative importance of (Bi) ORs with *β*-Sn versus Cu_6_Sn_5_ and no OR, the proportion of (Bi) particles with ORs to different phases are visualised as a function of thermal history in Fig. 12d. The column charts in Fig. 12d summarise the fractions of (Bi) particles with ORs to *β*-Sn, Cu_6_Sn_5_, Ag_3_Sn, and without an OR to any phase. For each group of (Bi), at least 300 (Bi) particles were analysed. For surface-precipitated (Bi) (right), over 80% of particles exhibited an OR with *β*-Sn, indicating a strong tendency for (Bi) particles to nucleate on the *β*-Sn free surface, and for (Bi) particles with an OR to *β*-Sn to prevail during surface coarsening. These (Bi) particles were distributed uniformly across the *β*-Sn matrix (Fig. 1b), indicating that nucleation occurred in the bulk *β*-Sn in these cases rather than at grain boundaries or interphase boundaries. A similar distribution was observed for bulk-precipitated (Bi) in thermally cycled joints (middle), with a lower fraction (~ 65%) of (Bi) particles with an OR to *β*-Sn. These (Bi) particles often shared interfaces with Cu_6_Sn_5_ or Ag_3_Sn particles (Fig. 1d), suggesting that (Bi) particles heterogeneously nucleated at the interfaces between *β*-Sn and IMC particles to reduce the energy barrier. For both of these thermally cycled groups, nearly no (Bi) particles had an OR with Cu_6_Sn_5_. The higher preference for surface-precipitated (Bi) to have an OR with *β*-Sn (than bulk (Bi) particles) is likely to be related to the ~ 30% volumetric expansion when (Bi) precipitates in *β*-Sn, since surface precipitation allows (Bi) to protrude at the free surface and, thus, reduce the misfit strain energy. This is demonstrated in SI-Fig. 4 in the Supplementary Information.

In the bulk of soldered-then-stored joints (left), ~ 7% of (Bi) particles exhibited an OR with Cu_6_Sn_5_, and ~ 50% of (Bi) particles exhibited an OR with *β*-Sn. This shows that (Bi) preferentially nucleates in the *β*-Sn matrix rather than on Cu_6_Sn_5_ particles during room-temperature ageing after soldering, although both can occur. This preference for precipitation in *β*-Sn is consistent with Table 3, where the best matching (Bi)/*β*-Sn planes in the ORs have a disregistry of 6.5% and 5.2% whereas the best matching (Bi)/Cu_6_Sn_5_ planes have a higher (worse) disregistry of 7.8% or 9.8%. The lack of a measured OR between (Bi) and Ag_3_Sn indicates that the atomic match between available planes in (Bi) and Ag_3_Sn is worse than for (Bi) on *β*-Sn or Cu_6_Sn_5_. The majority of (Bi) particles having an OR with *β*-Sn shared interfaces with IMC particles (Fig. 1c), indicating that these particles may heterogeneously nucleate at the interfaces between *β*-Sn and IMC particles.

While the focus of this paper has been on the effects of thermal history on the crystallographic aspects of (Bi) precipitation and the differences between surface and bulk (Bi) precipitation, the results also have significance for the industrial use of Sn–Ag–Cu–Bi solders containing the (Bi) phase. Bi is typically added to Sn–Ag–Cu solders to increase strength through solid solution strengthening and, when the Bi concentration is high enough, additionally through precipitation strengthening by (Bi). Since the initially-nanoscale (Bi) precipitates became particles several μm in size after > 6 years of room-temperature storage (Fig. 1 and Table 1), this work has shown that (Bi) precipitates in the bulk solder are relatively unstable to room-temperature coarsening, with highly over-aged precipitates and the accumulation of (Bi) around Cu_6_Sn_5_ and Ag_3_Sn particles. Since coarse and widely spaced precipitates are generally poor strengtheners compared with the fine nanoscale precipitates present in the early stages of ageing, it is expected that the over-ageing at room temperature will lead to increased creep rate and plastic deformation of Sn-2.25Ag-0.5Cu-6Bi in service and, therefore, to performance degradation. This work, therefore, also highlights the need for future detailed studies linking the ageing of (Bi) precipitates to creep and fatigue resistance in Sn–Ag–Cu–Bi solders. This microstructure evolution at room temperature should be considered when using solders strengthened by (Bi) precipitates in applications where electronics are unused for years, either at the start of their life or intermittently during service. At the same time, given sufficient time above the (Bi) solvus line, it is expected that all (Bi) precipitates will redissolve into the *β*-Sn phase and, therefore, the over-ageing should be reversible. For example, once an electronic device is operated after an extended period of room-temperature storage, Joule heating can raise the temperature above the solvus line (Fig. 1f) and dissolve the (Bi) precipitates. However, the larger the precipitates the longer the required dissolution time will be and the temperature–time profile due to cyclic Joule heating can be highly variable. Thus, to determine whether complete reversibility of the over-ageing can be achieved in a given package, the dissolution time would need to be determined for the specific Joule heating conditions of the specific package.

## Conclusions

This study has revealed insights into the crystallographic aspects of (Bi) precipitates in Sn-2.25Ag-0.5Cu-6Bi BGA solder joints after long-term (> 6 years) room-temperature ageing following soldering and following harsh accelerated thermal cycling from −55/125 °C. It has been found that the behaviour of (Bi) precipitates in the bulk is markedly different to surface precipitation after polishing, and the crystallographic aspects of (Bi) precipitates also depend on whether the electronic package was previously thermally cycled or not. The key findings can be summarised as follows:

1. Five orientation relationships (ORs) were measured between (Bi) particles and *β*-Sn or Cu_6_Sn_5_ that can be written as follows.
  OR1 $$\left\{ {100} \right\}_{{\beta {\mathrm{Sn}}}} \parallel \left\{ {01\bar{1}2} \right\}_{{\left( {{\mathrm{Bi}}} \right)}} {\text{and }}\langle 001\rangle _{{\beta Sn}} \parallel \langle 2\bar{2}01\rangle _{{\left( {{\mathrm{Bi}}} \right)}}$$
  OR2a $$\left\{ {100} \right\}_{{\beta {\mathrm{Sn}}}} \parallel \left\{ {01\bar{1}2} \right\}_{{\left( {{\mathrm{Bi}}} \right)}} and\langle 011\rangle _{{\beta {\mathrm{Sn}}}} \parallel \langle 2\bar{2}01\rangle _{{\left( {{\mathrm{Bi}}} \right)}}$$
  OR2b $$\left\{ {100} \right\}_{{\beta {\mathrm{Sn}}}} \parallel \left\{ {\bar{1}012} \right\}_{{\left( {{\mathrm{Bi}}} \right)}} {\mathrm{and}}\langle 011\rangle _{{\beta {\mathrm{Sn}}}} \parallel \langle 2\bar{2}01\rangle _{{\left( {{\mathrm{Bi}}} \right)}}$$
  ORa $$\left\{ {01\bar{1}1} \right\}_{{{\mathrm{Cu6Sn5}}}} \parallel \left\{ {01\bar{1}2} \right\}_{{\left( {{\mathrm{Bi}}} \right)}} {\mathrm{and}}\langle 1\bar{1}01\rangle _{{{\mathrm{Cu6Sn5}}}} \parallel \langle 2\bar{2}01\rangle _{{\left( {{\mathrm{Bi}}} \right)}}$$
  ORb $$\left\{ {01\bar{1}1} \right\}_{{{\mathrm{Cu6Sn5}}}} \parallel \left\{ {\bar{1}012} \right\}_{{\left( {{\mathrm{Bi}}} \right)}} {\mathrm{and}}\langle 1\bar{1}01\rangle _{{{\mathrm{Cu6Sn5}}}} \parallel \langle 2\bar{2}01\rangle _{{\left( {{\mathrm{Bi}}} \right)}}$$
2. OR1 has eight orientation variants for (Bi) precipitating in *β*-Sn and all eight were observed in some areas. OR2 can be divided into OR2a and OR2b, each with eight orientation variants for (Bi), and 10 of these 16 (Bi) orientations were observed together in some areas. The multiple alignments of OR2 (Bi) precipitates, e. g. with an ${\langle 2\bar{2}01\rangle }_{\left(\mathrm{Bi}\right)}$ parallel with any one of the four different $\left\langle {011} \right\rangle_{{\beta {\mathrm{Sn}}}}$ axes in a single *β*-Sn grain, have been shown to be consistent with these orientation variants.
3. The plate morphologies of (Bi) particles with OR1 and OR2 are in good agreement with the unstrained interfacial coherency at the habit planes, where the largest habit plane of a (Bi) particle tends to be the (Bi)/*β*-Sn interface with the lowest planar disregistry. Specifically, the largest habit plane was $\left\{ {100} \right\}_{{\beta {\mathrm{Sn}}}} \parallel \left\{ {01\bar{1}2} \right\}_{{{\mathrm{Bi}}}}$ for OR1 and $\left\{ {011} \right\}_{{\beta {\mathrm{Sn}}}} \parallel \left\{ {01\bar{1}2} \right\}_{{{\mathrm{Bi}}}}$ for OR2.
4. The angular mismatch caused by the 87.6° angle between adjacent $\left\{ {01\bar{1}2} \right\}$ of (Bi) and 90° angle between adjacent $\left\{100\right\}$ and $\left\{001\right\}$ of *β*-Sn also affects (Bi) plate morphologies, where in OR1, the parallel $\left\{ {100} \right\}_{{\beta {\mathrm{Sn}}}} \parallel \left\{ {01\bar{1}2} \right\}_{{{\mathrm{Bi}}}}$ tends to be larger than the near-parallel $\left\{ {010} \right\}_{{\beta {\mathrm{Sn}}}} /\left\{ {01\bar{1}2} \right\}_{{{\mathrm{Bi}}}}$ interface with the angular mismatch.
5. Unlike surface-precipitated (Bi) which predominantly exhibits OR1 after coarsening, bulk-precipitated (Bi) had both OR2 and OR1 with a preference for OR2 (over twice as many particles per unit area with OR2 as with OR1).
6. The ORs between (Bi) and Cu_6_Sn_5_ particles were only observed for (Bi) in the bulk of soldered-then-stored joints.
7. During long-term room-temperature storage (i.e. natural ageing) of 7 years after soldering or 6 years after thermal cycling, coarsening resulted in both large (> 1 μm) widely spaced (Bi) particles and the accumulation of (Bi) around IMC particles, which are expected to lead to an increased creep rate and performance degradation. This should be considered when using (Bi) precipitate-strengthened solders in applications where electronics are unused for years, either at the start of their life or intermittently during service.

## Electronic supplementary material

Below is the link to the electronic supplementary material.

Supplementary file1 (DOCX 3696 kb)

## Appendix

### Orientation variants of (Bi) in the ORs with hexagonal ***η***-Cu_6_Sn_5_

See Fig. 13

### Orientation variants of (Bi) in the ORs with monoclinic ***η***’-Cu_6_Sn_5_

See Fig. 14
