# Supplementary material for: Effects of thermal cycling and room-temperature ageing on bismuth precipitates in Sn-Ag-Cu-Bi solder joints
Source: J Mater Sci. 2026 Apr 28;61(23):16674–704. doi: 10.1007/s10853-026-12718-8 (PMC13153040; doi:10.1007/s10853-026-12718-8)
Supplement: Supplementary file 1 — Supplementary file1 (DOCX 3696 kb) [file 10853_2026_12718_MOESM1_ESM.docx]

# Supplementary Information

Effects of Thermal Cycling and Room Temperature Aging on Bismuth Precipitates in Sn-Ag-Cu-Bi Solder Joints

C.L. Hsieh^1^, R.J. Coyle ^2^, J.W. Xian^1,3^, C.M. Gourlay^1^

^1^ Department of Materials, Imperial College London, London. SW7 2AZ. UK

^2^ Nokia Bell Labs, Murray Hill, NJ, USA

^3^ School of Materials Science and Engineering, Dalian University of Technology, Dalian, China

**Depression in β-Sn liquidus temperature due to the addition of Bi:**

| 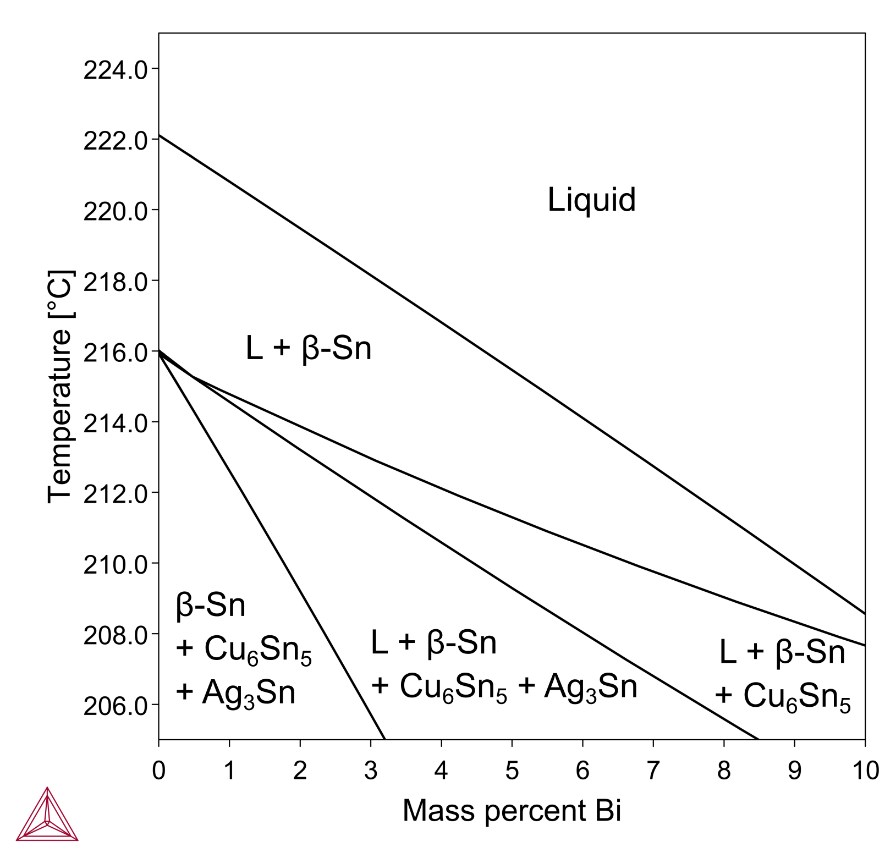  SI-Figure 1 The depression in β-Sn liquidus temperature due to the addition of Bi. (97.25-x)Sn-2.25Ag-0.5Cu-xBi isopleth showing the β-Sn liquidus is lowered by ~8 K by adding 6wt.% Bi. Calculations were performed in Thermo-Calc with the NIST solder database [1]. |
| --- |

**Similar (Bi) particle features between 192CABGA and 84CTBGA packages:**

| 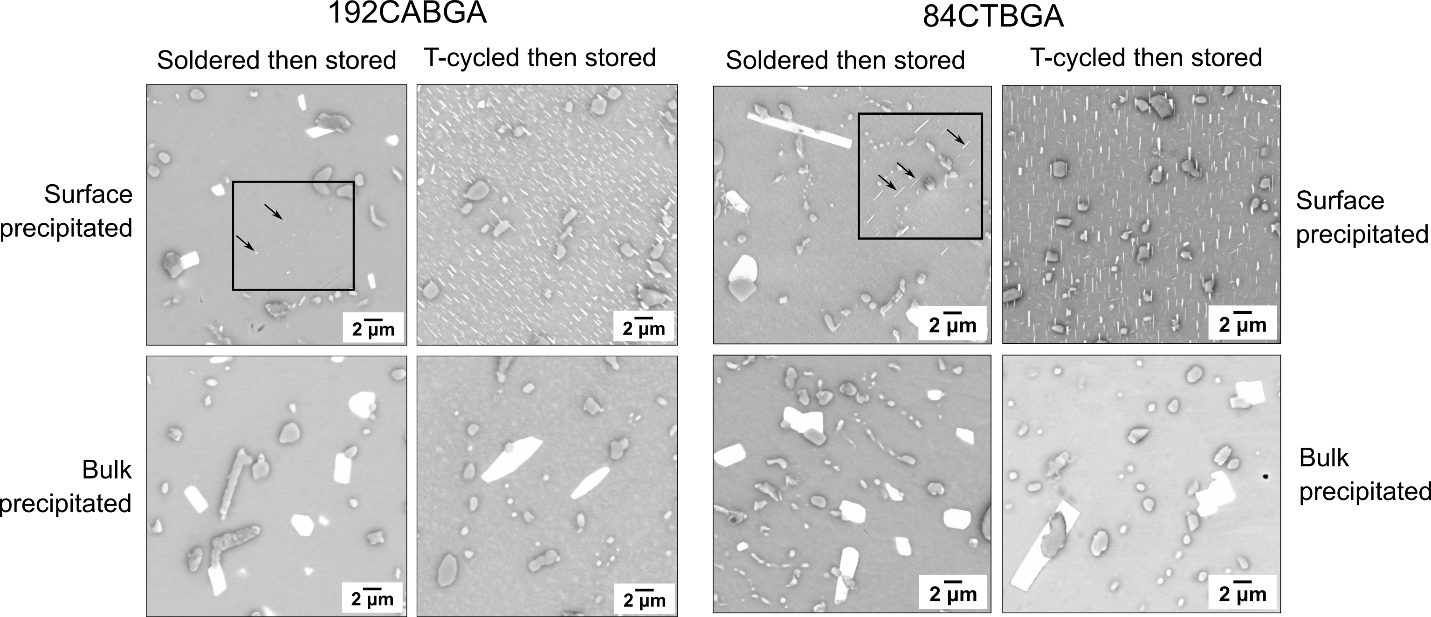  SI-Figure 2 Dependence of (Bi) particle features on their location in solder joints and their thermal history for both 192CABGA (left) and 84CTBGA (right) packages. Note that there is no difference in (Bi) particle feature between both packages. |
| --- |

**Procedures for estimating the lattice spacings of β-Sn with Bi:**

| 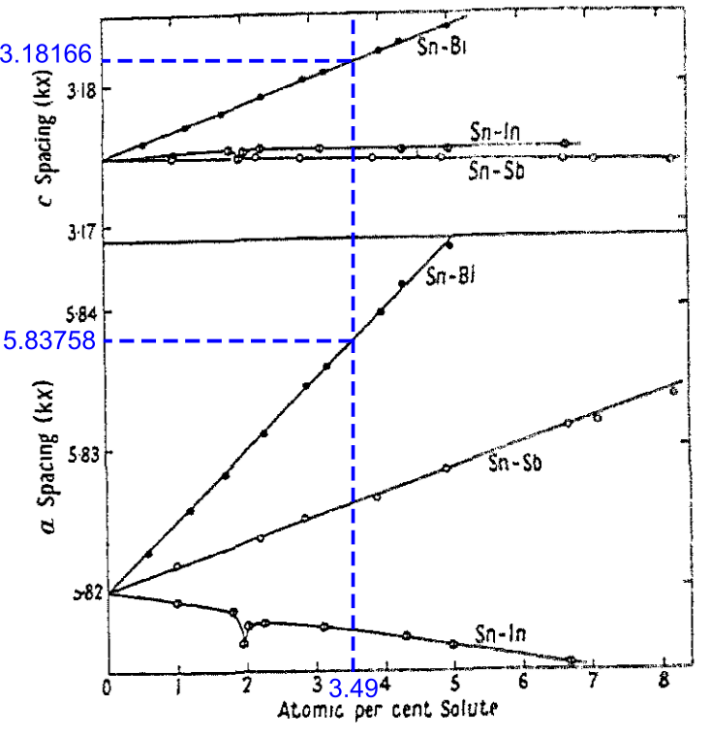  SI-Figure 3 The change of β-Sn lattice spacings with the atomic percent of Bi, Sb, and In in β-Sn as reproduced from Ref. [2]. The slopes of the fitted lines indicate the extent of lattice spacing change per unit atomic percent of solute. To measure the slopes of the fitted lines, a random atomic percent solute value was selected from the plot, e.g. 3.49 at.% as indicated by the dashed line. Then, the c-spacing and a-spacing corresponding to 3.49 at.% Bi can be digitised from the plot using ImageJ. The a-spacing and c-spacing of β-Sn with 0 at.% Bi were 5.82 Å and 3.175 Å [2], so the slopes are measured to be 0.00504 and 0.00191 for a-spacing and c-spacing. Therefore, the relationships between a-spacing/c-spacing and at.% of Bi can be expressed as:  $a=0.00504*C_{solute}+5.82 (Å)$  $c=0.00191*C_{solute}+3.175 (Å)$  where $C_{solute}$ is the concentration of Bi in β-Sn in atomic percent. As the lattice spacing of pure β-Sn was refined in a later study [3], the equations can be further modified as:  $a=0.00504*C_{solute}+5.831 (Å)$  $c=0.00191*C_{solute}+3.182 (Å)$  These equations are used for estimating the lattice spacings of β-Sn matrix containing Bi. |
| --- |

**Volumetric strain associated with the precipitation of (Bi) from β-Sn:**

| 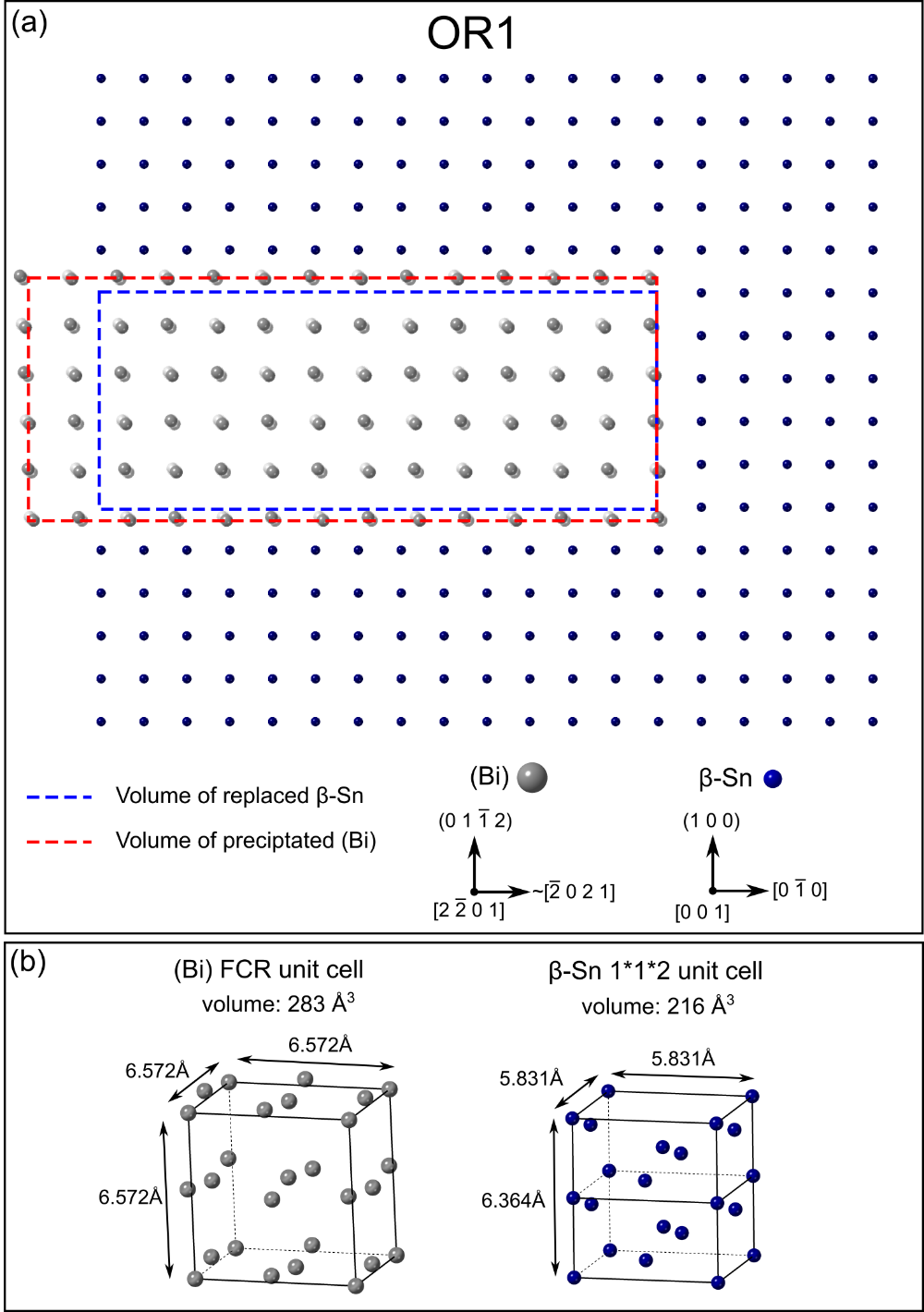  SI-Figure 4 Volumetric misfit strain between (Bi) precipitates and the β-Sn matrix. (a) Atomic arrangement of a (Bi) particle precipitating from a β-Sn matrix and sharing OR1 with the β-Sn, projected along ${[001]}_{\beta Sn}{\parallel[2\bar{2}01]}_{\left( Bi \right)}$. The red dashed box represents the volume of the precipitated (Bi), and the blue dashed box represents the volume that used to be β-Sn. This figure is plotted for unstrained crystals without dislocations. (b) A (Bi) face-centered rhombohedral (FCR) unit cell and β-Sn 1*1*2 tetragonal unit cells oriented together according to OR1. Assuming unstrained crystals without dislocations, Sn atoms are replaced by an equal number of Bi atoms when (Bi) precipitates from β-Sn. A BCT unit cell of β-Sn contains 4 Sn atoms, while a FCR unit cell of (Bi) contains 8 Bi atoms. Thus, when (Bi) precipitates from β-Sn, one FCR unit cell of (Bi) takes the place of two tetragonal unit cells of β-Sn. The difference in volume between the two phases can be expressed as ΔV= n(V_(Bi)_ - 2V_βSn_) and the misfit strain can be calculated by ε_vol_ = ΔV/2V_βSn_. Based on the structures in Table 1, the FCR unit cell volumes of (Bi) and β-Sn are 283.07 Å^3^ and 108.19 Å^3^ respectively. Thus, the misfit strain ε_vol_ between (Bi) and β-Sn is calculated to be 30.8%. |
| --- |

| 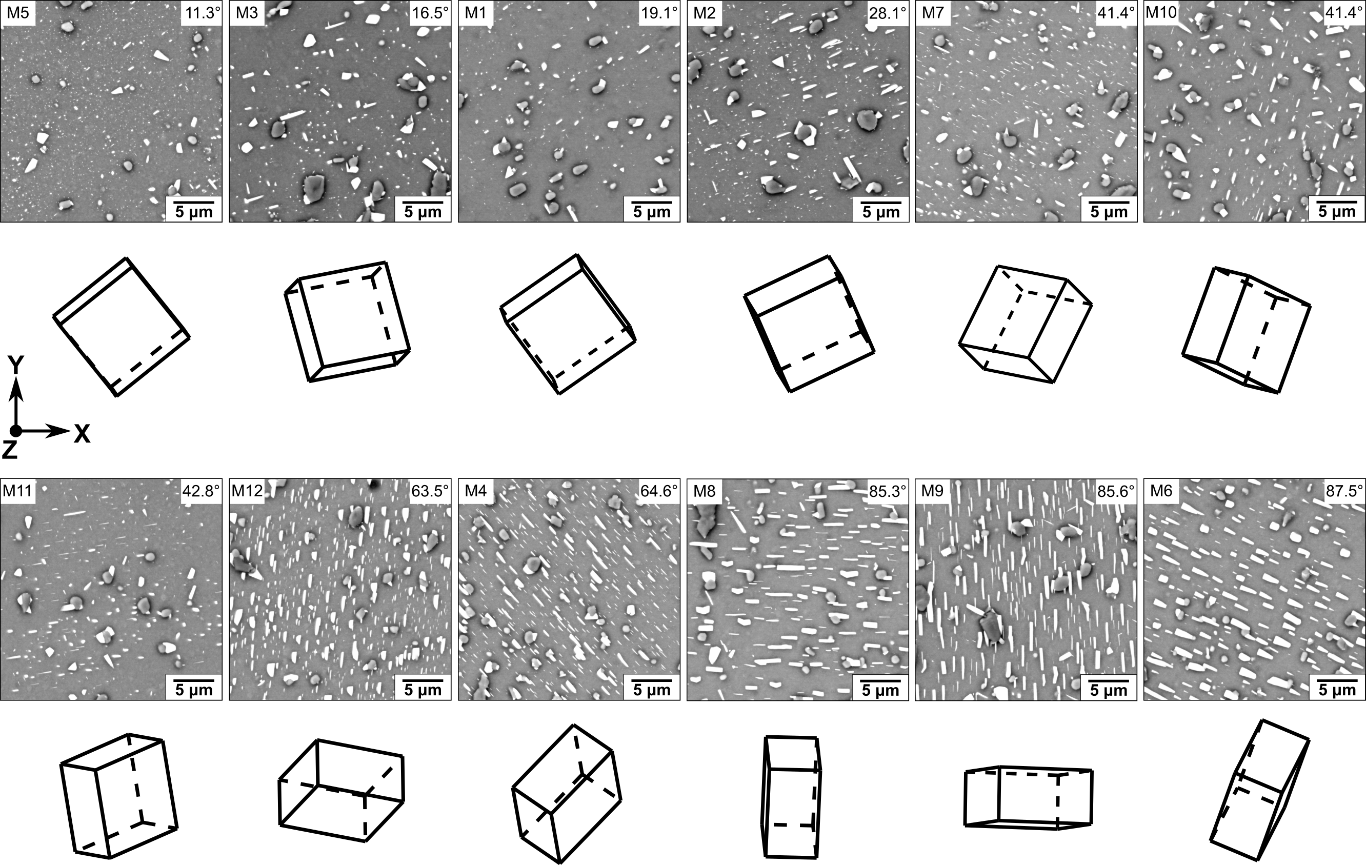  SI-Figure 5 BSE images of all 12 joints in Figure 9(b) taken 67 days after polishing. The images are ordered based on the angle between $\left\langle001 \right\rangle_{\beta Sn}$ and the surface normal, Z, which is labelled at the top right of each BSE image. The β-Sn unit cell wireframes beneath each BSE image were plotted from the Euler angles measured by EBSD and indicate the β-Sn orientation of each joint. Note that $\left\langle001 \right\rangle_{\beta Sn}$ is the short axis of the unit cell. |
| --- |

**Identification of (Bi) phase with EDS and EBSD:**

| 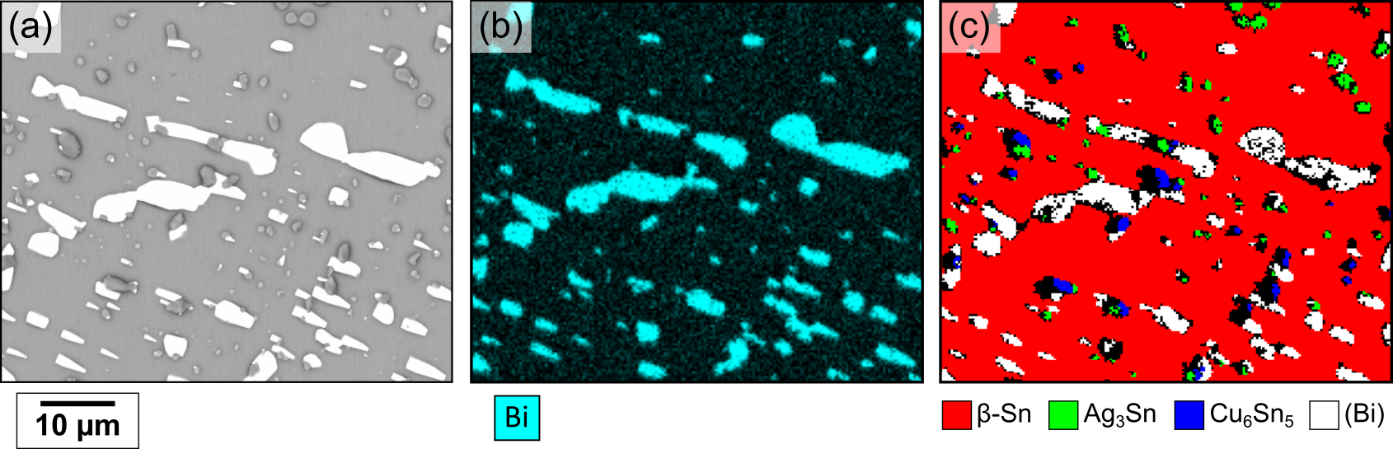  SI-Figure 6 (a) SEM BSE image, (b) EDS Bi map, and (c) EBSD phase map of a region in a thermally-cycled-then-stored 84CTBGA joint. (Bi) phase appeared as brightest particles in BSE micrographs due to the significantly higher atomic number of Bi (84) compared to Sn (50) and Ag (47). The elemental composition of the brightest particles can be further confirmed as pure bismuth by EDS. |
| --- |

**Similar distributions in IPFs between (Bi)/β-Sn OR2a and OR2b:**

| 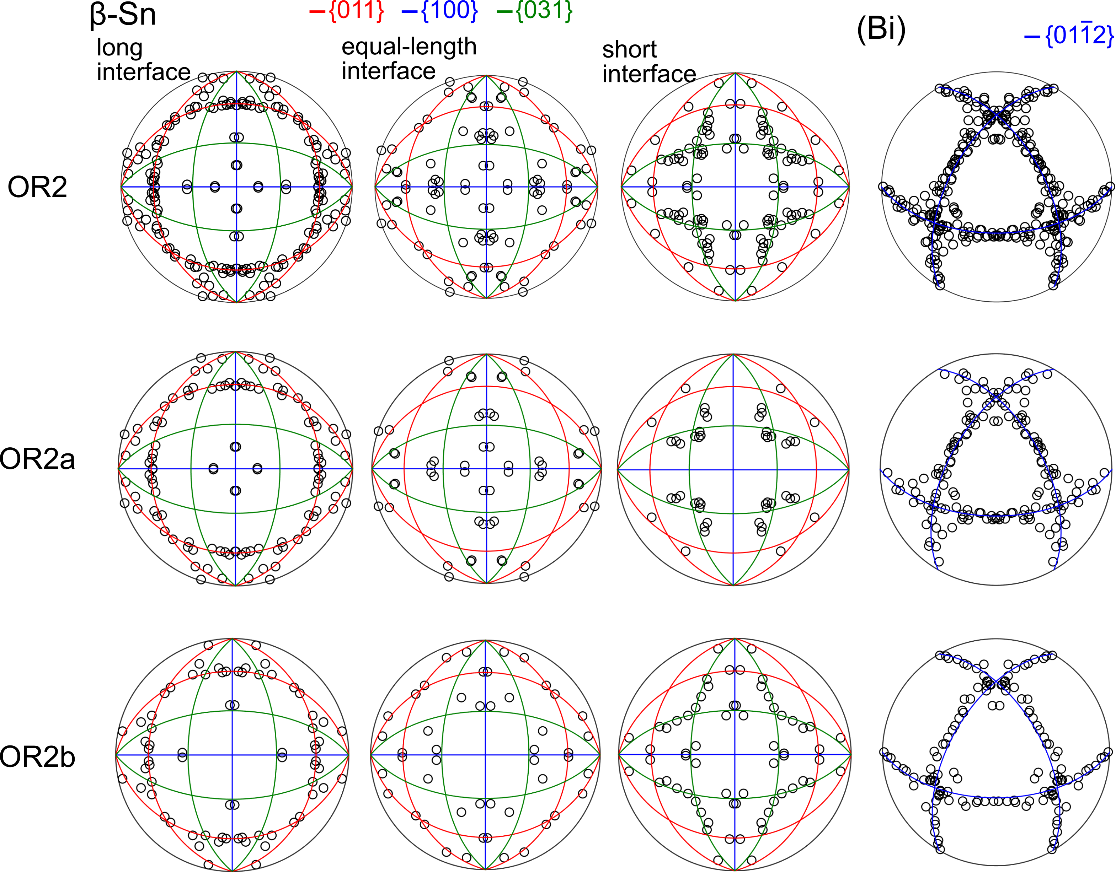  SI-Figure 7 Inverse pole figure (IPF) analysis of (Bi)/β-Sn interfaces with OR2 (combined with OR2a and OR2b) (top), OR2a (middle) and OR2b (bottom). IPFs of β-Sn and (Bi) summarise the lattice directions lying in (Bi)/β-Sn interface planes measured from BSE images. For the IPF of β-Sn, the data are separately plotted for long interfaces, equal-length interfaces, and short interfaces. Note that both OR2a and OR2b display very similar distribution of IPFs. |
| --- |

**Supplementary references:**

[1] National Institute of Standards and Technology, “Phase Diagram & Computational Thermodynamics.” Accessed: Jul. 16, 2024. [Online]. Available: https://www.metallurgy.nist.gov/phase/solder/agcusn.html

[2] J. A. Lee and G. V. Raynor, “The lattice spacings of binary tin-rich alloys,” *Proceedings of the Physical Society. Section B*, vol. 67, no. 10, pp. 737–747, 1954, doi: 10.1088/0370-1301/67/10/301.

[3] P. Cucka and C. S. Barrett, “The crystal structure of Bi and of solid solutions of Pb, Sn, Sb and Te in Bi,” *Acta Crystallogr*, vol. 15, no. 9, pp. 865–872, Sep. 1962, doi: 10.1107/s0365110x62002297.
